# Distinct Persistence Fate of Mycobacterium tuberculosis in Various Types of Cells

**DOI:** 10.1128/mSystems.00783-21

**Published:** 2021-08-17

**Authors:** Xi Chen, Xiaojian Cao, Yingying Lei, Aikebaier Reheman, Wei Zhou, Bing Yang, Weipan Zhang, Weize Xu, Shuang Dong, Rohit Tyagi, Zhen F. Fu, Gang Cao

**Affiliations:** a State Key Laboratory of Agricultural Microbiology, College of Veterinary Medicine, Huazhong Agricultural University, Wuhan, China; b Bio-Medical Center, Huazhong Agricultural University, Wuhan, China; c College of Animal Science, Tarim University, Xinjiang, China; University of Delhi

**Keywords:** phagosome maturation arrest, *Mycobacterium tuberculosis*, autophagy, *itgb3*, persistence

## Abstract

Mycobacterium tuberculosis can invade different cells with distinct persistence fates because cells are equipped with different host restriction factors. However, the underlying mechanisms remain elusive. Here, we infected THP1 and Raw264.7 macrophages cell lines, A549 epithelial cell line, and hBMEC and bEnd.3 endothelial cell lines with M. tuberculosis and demonstrated that M. tuberculosis significantly inhibited lysosome acidification in THP1, hBMEC, A549, and Raw264.7 cells, while, in bEnd.3 cells, M. tuberculosis was mainly delivered into acidified phagolysosomes and auto-lysosomes. The systematic gene profile analysis of different cells and intracellular M. tuberculosis showed that the phagosome autophagy-pathway-related genes *itgb3* and *atg3* were highly expressed in bEnd.3 cells. Knockdown of these genes significantly increased the number of viable intracellular M. tuberculosis bacilli by altering phagosomal trafficking in bEnd.3 cells. Treatment with *itgb3* agonist significantly decreased M. tuberculosis survival *in vivo*. These findings could facilitate the identification of anti-M. tuberculosis host genes and guide M. tuberculosis-resistant livestock breeding.

**IMPORTANCE** As an intracellular pathogen, Mycobacterium tuberculosis could avoid host cell immune clearance using multiple strategies for its long-term survival. Understanding these processes could facilitate the development of new approaches to restrict intracellular M. tuberculosis survival. Here, we characterized the detailed molecular events occurring during intracellular trafficking of M. tuberculosis in macrophage, epithelial, and endothelial cell lines and found that ITGB3 facilitates M. tuberculosis clearance in endothelial cells through altering phagosomal trafficking. Meanwhile, the treatment with ITGB3 agonist could reduce bacterial load *in vivo*. Our results identified new anti-M. tuberculosis restriction factors and illuminated a new anti-M. tuberculosis defense mechanism.

## INTRODUCTION

The etiologic agent of tuberculosis (TB), Mycobacterium tuberculosis, is a facultative intracellular pathogen. In 2019, TB caused an estimated 1.4 million deaths and 10 million new cases were reported worldwide (1). As an aerosol transmission pathogen, M. tuberculosis is first recognized and undergoes phagocytosis by alveolar macrophages inside the lungs. After engulfment, M. tuberculosis can induce the formation of cellular mass known as granuloma and persist in it, leading to the latent phase of the disease. In the active phase, M. tuberculosis has the ability to induce phagocytosis in the nearby cells for its expansion. In this expansion, it can invade nonimmune cells like epithelial and endothelial cells to find new avenues for spreading. When internalized into nonimmune cells, M. tuberculosis can replicate in epithelial cells but persist in brain microvascular endothelial cells (BMECs).

The ultimate killing of M. tuberculosis by host cells depends on multiple factors, such as the acidification of lysosomes, the process of autophagy, the delivery of antimicrobial peptides, and other restriction factors (2). Understanding these processes may greatly facilitate the development of new approaches to restrict intracellular M. tuberculosis survival. A few genome-wide studies have identified the host anti-M. tuberculosis restriction factors (3, 4). Granulocyte-macrophage colony-stimulating factor (GM-CSF) produced by T cells, macrophages, or alveolar epithelial cells is vital for the host to inhibit intracellular M. tuberculosis growth (5, 6). However, the restriction factors and their molecular defense mechanisms against M. tuberculosis in different cells are still not well understood.

The pathogenicity of microorganisms is often related to their capacity to survive and replicate within their hosts. While the phagosome-lysosome pathway functions effectively as the front line to kill the invading M. tuberculosis, the bacteria have evolved various elements of molecular machinery to escape from this clearance. M. tuberculosis avoids lysosomal fusion through alteration of endocytic trafficking, resulting in privileged multiplicative niches. In this scenario, it has been demonstrated that the phagosome markers Rab5 and Rab7, but not the lysosome markers LAMP and cathepsin, rapidly accumulate in the mycobacterial endosomal membrane in macrophages, epithelial cells, and endothelial cells (7–9). It has also been reported that the lack of acidification in the mycobacteria-containing phagosomes, due to the absence of lysosomal acid proteases on the phagosomal membranes, facilitates the survival of intracellular M. tuberculosis (10).

During the process of altering endocytic trafficking, ESAT6 secreted from M. tuberculosis can breach the phagosome membrane and lead to the translocation of M. tuberculosis into the cytosol for its survival (11, 12). The permeabilization of the phagosomal membrane results in phagosomal and cytoplasmic mixing and allows extracellular mycobacteria DNA to access host cytosolic receptors, such as cGAS (13, 14). The binding of mycobacteria DNA with cGAS induces type I interferon, which paradoxically facilitates M. tuberculosis replication and activates ubiquitin-mediated autophagy to restrict M. tuberculosis replication (15). Autophagy is involved in the innate defense against invading intracellular pathogens by sequestering them into autophagosomes and delivering them to the lysosomes (16, 17). M. tuberculosis interferes with autophagy-related pathways via various bacterial proteins. Dimycocerosate (DIM), a major virulence factor of M. tuberculosis, prevents phagosomal damage-independent autophagy and limits the acidification of LC3-positive M. tuberculosis compartments (18). Despite extensive documentation of the multiple functional intracellular pathogen-killing mechanisms in different cells, the underlying molecular mechanisms of M. tuberculosis clearance in different cells remains elusive.

The aim of this study is, therefore, to investigate the persistent fate and the underlying mechanisms of M. tuberculosis in different types of cells. By combining cell biology and molecular biology techniques, we characterized the detailed molecular events occurring during intracellular trafficking of M. tuberculosis and identified restriction factors in different types of host cells.

## RESULTS

### Distinct survival and replication fate of intracellular M. tuberculosis in different types of host cells.

**FIG 1:**
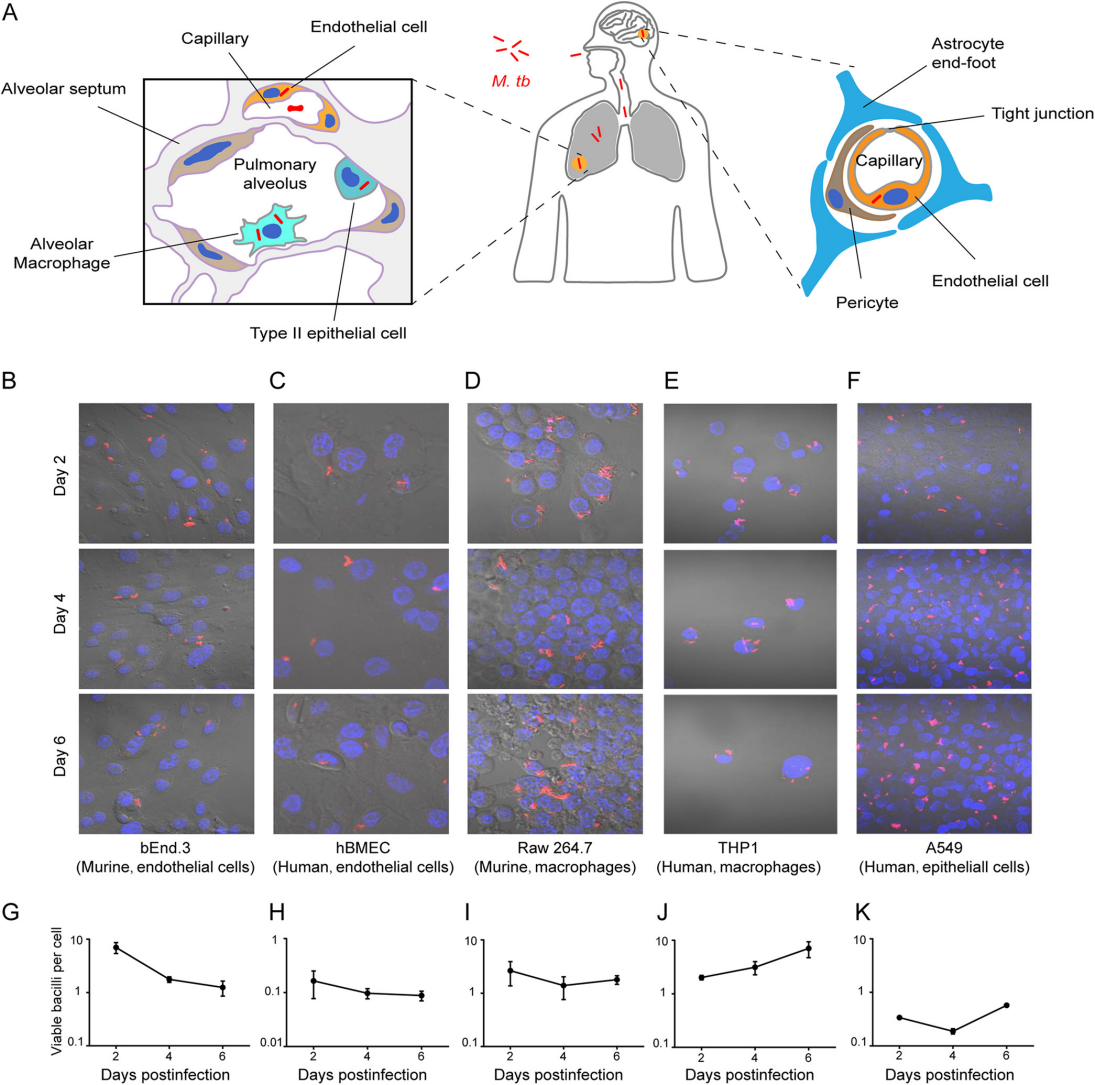
The distinct fate of M. tuberculosis in different cell lines. (A) Schematic of infection and transmission of M. tuberculosis in different organs and cells. (B to F) Visualization of M. tuberculosis-infected cells. Cells were seeded in a 24-well plate and infected with M. tuberculosis for 6 h and then imaged with confocal microscopy at the indicated time points. (G to K) The number of viable bacilli per cell. Cells were seeded in a 24-well plate and infected with M. tuberculosis for 6 h and then lysed for viable intracellular bacilli counting at the indicated time points. At the same time, cells were detached for cell counting. The number of viable bacilli per cell was calculated by total CFU/total number of live cells. All experiments were performed in triplicate and repeated at least three times. Data are presented as mean ± SEM.

After being inhaled by a human being, M. tuberculosis can locate to the pulmonary region and invade alveolar macrophages and type II epithelial cells. M. tuberculosis can break down the epithelial-endothelial barrier and cause extra-pulmonary tuberculosis. For example, it infects brain microvascular endothelial cells and disrupts the blood-brain barrier to cause central nervous system tuberculosis (Fig. 1A). To investigate the survival and replication fate of M. tuberculosis in different types of cells, an intracellular bacteria viability assay was carried out in endothelial cells (bEnd.3 and hBMEC), macrophages (Raw 264.7 and THP1), and epithelial cells (A549). First, the viability of the infected cells was examined on days 2, 4, and 6 postinfection. As shown in Fig. 1B to F, the persistence of M. tuberculosis strain H37Ra had a profound influence on the growth of THP1 cells, but less so on bEnd.3, hBMEC, Raw 264.7, and A549 cells. The numbers of bEnd.3, hBMEC, Raw 264.7, and A549 cells, but not THP1 cells, were increased over time (Fig. S1A to E in the supplemental material).

Next, we investigated the viability of intracellular M. tuberculosis. Our results showed that the total number of viable M. tuberculosis H37Ra bacilli decreased in bEnd.3, THP1, and Raw 264.7 cells over time, but increased in hBMECs and A549 cells (Fig. S1F to J). Similarly, M. tuberculosis strain H37Rv could replicate in neither bEnd.3 cells nor in hBMEC cells (Fig. S1K and L). Considering that the replication of M. tuberculosis could cause cell death and lead to the loss of M. tuberculosis during washing steps, the number of viable bacilli per cell was used to compare the survival and replication capability of M. tuberculosis in different cells. We found that at day 6, the number of viable M. tuberculosis per cell was significantly increased in comparison to day 2 in THP1 and A549 cells, but not in other examined cells (Fig. 1G to K). These data showed that M. tuberculosis could replicate in THP1 and A549 cells and could persist in hBMECs, bEnd.3, and Raw 264.7 cells.

### Distinct phagocytosis processes of M. tuberculosis in different types of cells.

**FIG 2:**
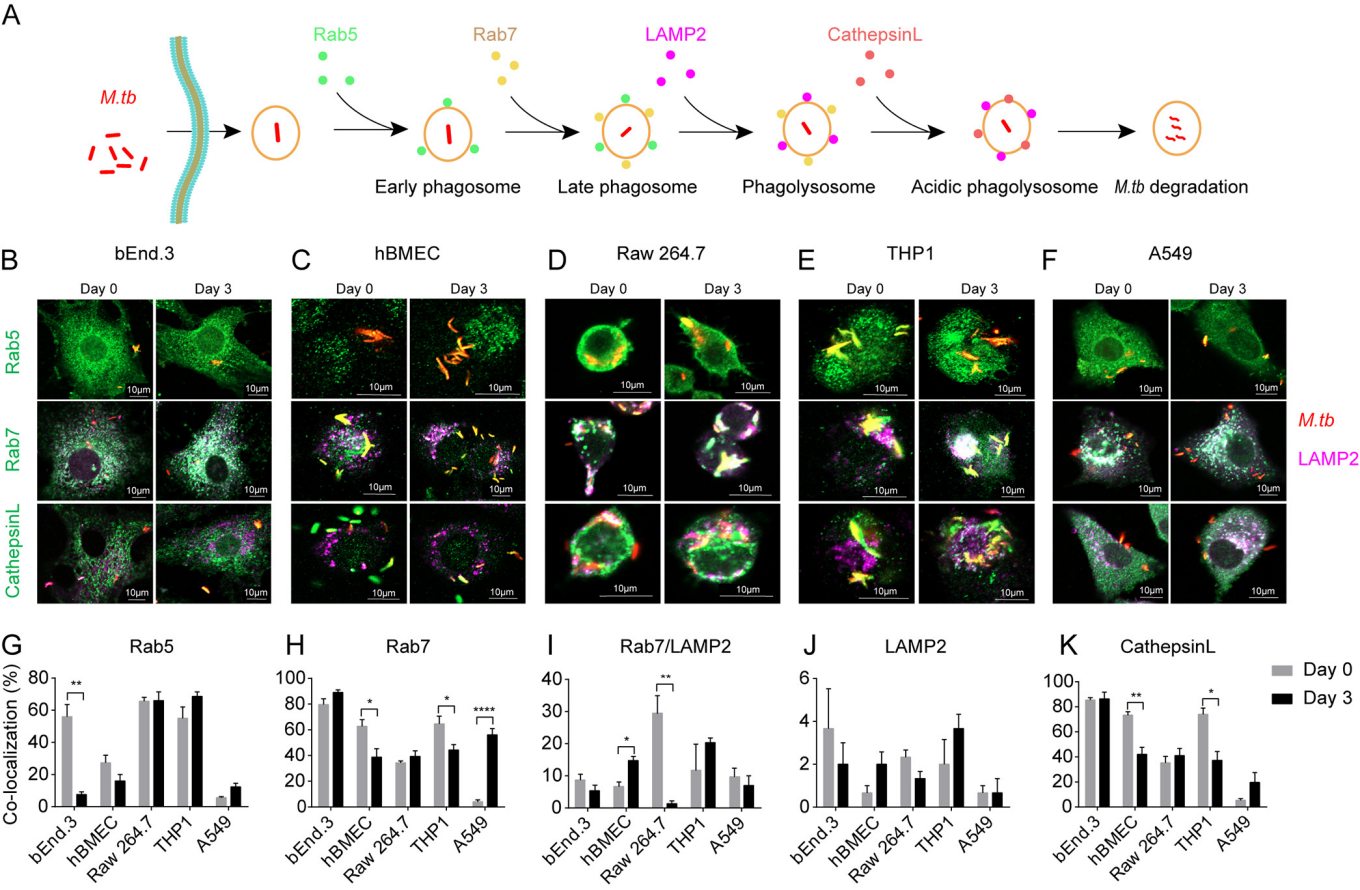
Phagocytosis trafficking of M. tuberculosis in different cell lines. (A) Schematic of M. tuberculosis phagocytosis trafficking in host cells. (B to F) Confocal microscopic images as indicated. Cells were seeded onto coverslips and infected with dsRed-expressing M. tuberculosis for 6 h. Cells were fixed at the indicated time points and incubated with rabbit anti-Rab5, rabbit anti-Rab7, or rabbit anti-cathepsin L and rat anti-LAMP2 antibodies, respectively. Alexa Fluor 488 goat anti-rabbit IgG (green) was used to detect Rab5, Rab7, and cathepsin L. Alexa Fluor 647 goat anti-rat IgG (purple) was used to detect LAMP2. (G to K) The colocalization of M. tuberculosis with Rab5 (G), Rab7 (H), Rab7/LAMP2 (I), LAMP2 (J), and cathepsin L (K) were quantified from 100 bacteria. All specimens were imaged with the confocal microscope and experiments were performed in triplicate and repeated two times. Data are presented as mean ± SEM and analyzed by unpaired *t* test with two-tailed *P* values, with asterisks indicating statistically significant differences: *, *P < *0.05; **, *P < *0.01; ***, *P < *0.001; ****, *P < *0.0001.

Next, we investigated the phagocytosis process of M. tuberculosis in various cell lines by co-staining with different phagosome markers, such as Rab5 and Rab7, or lysosome markers, such as LAMP2 and cathepsin L (Fig. 2A). As shown in Fig. 2B to F, M. tuberculosis H37Ra-containing vacuoles (MCVs) colocalized with the phagosome and lysosome markers. The association of MCVs with Rab5 was significantly decreased in bEnd.3 cells over time and maintained at a low level in both hBMEC and A549 cells, but maintained at a high level in Raw 264.7 and THP1 cells over time (Fig. 2G). During the infection with M. tuberculosis H37Rv, the association of MCVs with Rab5 was maintained at a low level over time in both hBMEC and THP1 cells (Fig. S2A and B). Further, we analyzed the colocalization of MCVs with the late phagosome marker (Rab7) and lysosome marker (LAMP2). MCVs were highly colocalized with Rab7 in bEnd.3 cells, but with a relatively low level in hBMEC, Raw 264.7, and THP1 cells. In A549 cells, the colocalization of MCVs with Rab7 significantly increased over time (Fig. 2H), whereas in both hBMEC and THP1 cells during infection with M. tuberculosis H37Rv, the association of MCVs with Rab7 was maintained at a low level over time (Fig. S2A and B). In Raw 264.7 cells, the colocalization of MCVs with both Rab7 and LAMP2 significantly decreased over time but was maintained at a low level in other examined cells (Fig. 2I). The colocalization of MCVs with LAMP2 was maintained at a low level in all examined cells over time (Fig. 2J, Fig. S2A and B).

As intracellular pathogens can be degraded in acidified lysosomes, we then analyzed the colocalization of MCVs with the lysosomal endopeptidase enzyme cathepsin L in all examined cells. The colocalization of MCVs with cathepsin L was maintained at a high level in bEnd.3 cells, but at a low level in Raw 264.7 and A549 cells over time. In hBMEC and THP1 cells, the colocalization of MCVs with cathepsin L was significantly decreased during M. tuberculosis H37Ra infection (Fig. 2K), while it was significantly increased during M. tuberculosis H37Rv infection over time (Fig. S2A and B). These data demonstrate that different M. tuberculosis strains underwent a distinct phagocytosis process in different cells. Overall, M. tuberculosis arrested more at the early phagosomal maturation stage in macrophages, while it arrested more at a late stage in epithelial cells. Of note, in murine endothelial cells, M. tuberculosis was differentially delivered into acidified phagolysosomes for degradation.

10.1128/mSystems.00783-21.1 FIG S1 CFUs and cell counts. Cells were seeded in a 24-well plate and infected with M. tuberculosis for 6 h. (A to E) Live cell numbers were counted after trypsinization. Total H37Ra (F to J) and H37Rv (K and L) CFUs were quantified as indicated. For intracellular viable M. tuberculosis counting, cells were lysed at the indicated time points. The lysates were plated onto Middle brook 7H11 agar after serial dilution and CFU/ml was analyzed. Data are presented as mean ± SD. All experiments were performed in triplicate. Download FIG S1, PDF file, 0.8 MB. Copyright © 2021 Chen et al. 2021 Chen et al. https://creativecommons.org/licenses/by/4.0/ This content is distributed under the terms of the Creative Commons Attribution 4.0 International license.

10.1128/mSystems.00783-21.2 FIG S2 Trafficking of M. tuberculosis H37Rv in hBMEC (A) and THP1 (B) cells. Cells were seeded onto coverslips and infected with dsRed-expressing M. tuberculosis H37Rv for 6 h. Cells were fixed at the indicated time points and incubated with rabbit anti-Rab5, rabbit anti-Rab7, rabbit anti-cathepsin L, or rabbit anti-LC3 and rat anti-LAMP2 antibodies, respectively. Alexa Fluor 488 goat anti-rabbit IgG (green) was used to detect Rab5, Rab7, cathepsin L, and LC3. Alexa Fluor 647 goat anti-rat IgG (purple) was used to detect LAMP2. The colocalization of MCVs with Rab5, Rab7, Rab7/LAMP2, cathepsin L (Cath), cathepsin L/LAMP2, LC3, LC3/LAMP2, and LAMP2 was quantified from 100 bacteria. All specimens were imaged with the Zeiss confocal microscope with ZEN software 2.3 and performed in triplicate. Data are presented as mean ± SEM and analyzed by unpaired *t* test with a two-tailed *P* value, with asterisks indicating statistically significant differences: * *P* < 0.05; ** *P* < 0.01; *** *P* < 0.001; **** *P* < 0.0001. Download FIG S2, PDF file, 38.1 MB. Copyright © 2021 Chen et al. 2021 Chen et al. https://creativecommons.org/licenses/by/4.0/ This content is distributed under the terms of the Creative Commons Attribution 4.0 International license.

### Distinct autocytosis processes of M. tuberculosis in different types of cells.

**FIG 3:**
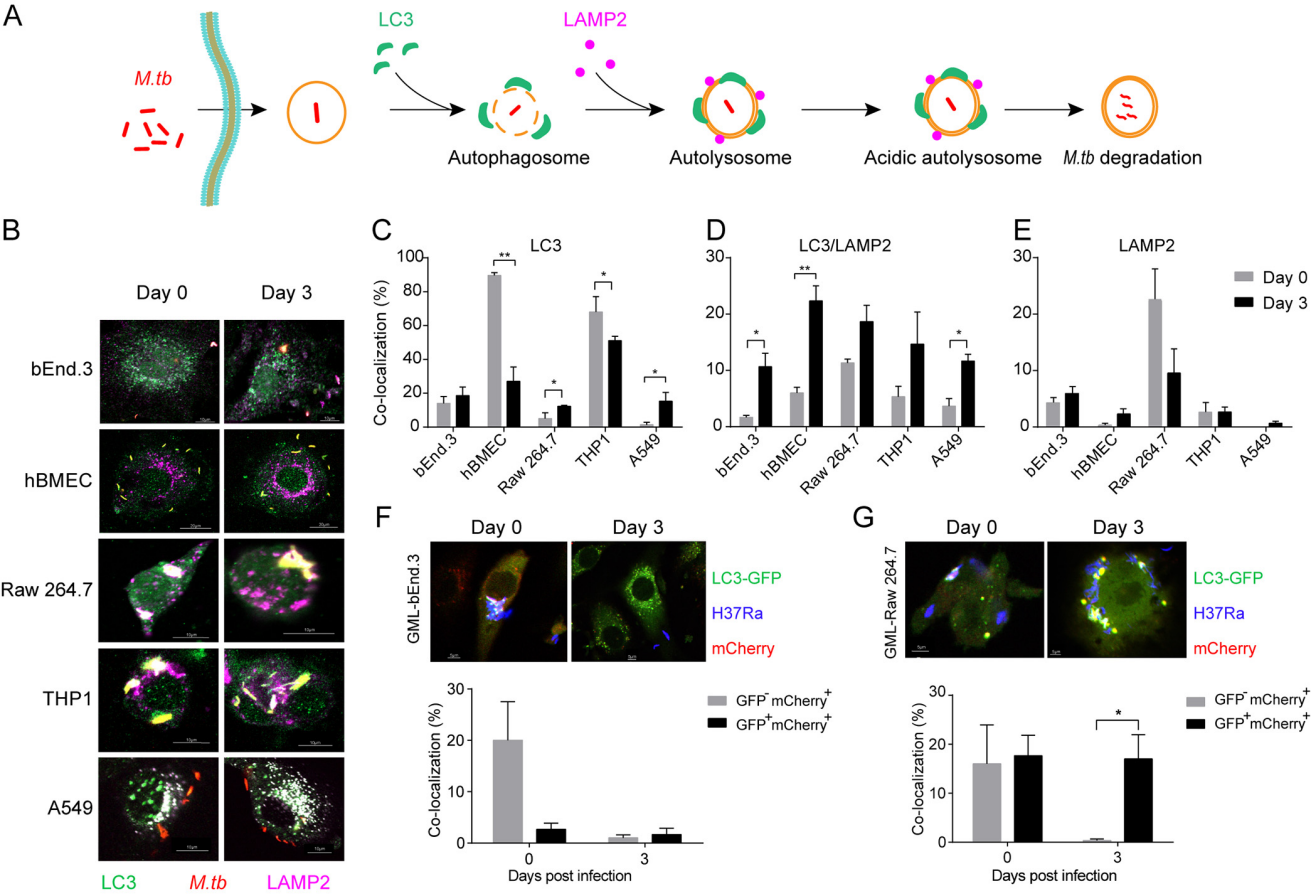
Autophagy trafficking of M. tuberculosis in different cell lines. (A) Schematic of M. tuberculosis autophagy trafficking pathway in host cells. (B) Confocal microscopic images as indicated. Cells were seeded onto coverslips and infected with dsRed-expressing M. tuberculosis for 6 h. Cells were fixed at the indicated time points and incubated with rabbit anti-LC3 and rat anti-LAMP2 antibodies. Alexa Fluor 488 goat anti-rabbit IgG (green) was used to detect LC3. Alexa Fluor 647 goat anti-rat IgG (purple) was used to detect LAMP2. (C to E) The colocalization of M. tuberculosis with LC3 (C), LC3/LAMP2 (D), and LAMP2 (E) was quantified from 100 bacteria. (F and G) For the acidification assay, GML-bEnd.3 (F) and GML-Raw 264.7 (G) cells were seeded onto coverslips and infected with blue fluorescent protein (BFP)-expressing M. tuberculosis. The colocalization of M. tuberculosis with mCherry alone or with both GFP and mCherry was quantified from 100 bacteria. All specimens were imaged with the confocal microscope and experiments were performed in triplicate and repeated three times. Data are presented as mean ± SEM and analyzed by unpaired *t* test with two-tailed *P* values, with asterisks indicating statistically significant differences: *, *P < *0.05; **, *P < *0.01; ***, *P < *0.001; ****, *P < *0.0001.

As autophagosomes could target disrupted MCVs toward lysosomes for degradation (Fig. 3A), we then analyzed the role of autophagy in M. tuberculosis survival in bEnd.3, hBMEC, Raw 264.7, THP1, and A549 cells. As shown in Fig. 3B, MCVs were associated with the autophagy marker LC3 in all the examined cells. In bEnd.3 and Raw 264.7 cells, the colocalization of MCVs with LC3 was maintained at a low level over time. The colocalization of MCVs with LC3 was significantly decreased in hBMECs and THP1 cells but significantly increased in A549 cells over time (Fig. 3C). During the infection with M. tuberculosis H37Rv, the association of MCVs with LC3 significantly increased over time in both hBMEC and THP1 cells (Fig. S2A and B).

For the colocalization of MCVs with both LC3 and LAMP2, no significant difference was observed in Raw 264.7 and THP1 cells, whereas, in bEnd.3 cells, hBMECs, and A549 cells, the colocalization of MCVs with both LC3 and LAMP2 was significantly increased (Fig. 3D). During M. tuberculosis H37Rv infection, a similar colocalization of MCVs with both LC3 and LAMP2 was observed in THP1 and hBMEC cells (Fig. S2A and B). In all examined cells, the colocalization of MCVs with LAMP2 was maintained at a low level over time (Fig. 3E, Fig. S2A and B). Further, a Western blot analysis was performed to examine autophagy by detection of Beclin-1 and LC3. As shown in Fig. S3, more Beclin-1 and matured LC3II were observed in M. tuberculosis-infected A549, hBMEC, Raw 264.7, and THP1 cells than in the the uninfected control cells. However, in M. tuberculosis-infected bEnd.3 cells, Beclin-1 and matured LC3II were decreased at day 3 postinfection. These data demonstrated that M. tuberculosis might block autophagosome fusion with lysosomes in bEnd.3, Raw 264.7 and A549 cells, but were delivered into auto-lysosome in THP1 and hBMEC.

Next, we investigated the acidification of auto-lysosomes during M. tuberculosis infection in different cells. To this end, GML-bEnd.3 and GML-Raw 264.7 cell lines stably expressing an auto-lysosome formation and the chimeric fusion reporter chimeric gene GFPph-mCherry-LC3 were constructed by CRISPR/Cas9-mediated recombination and employed to compare the lysosomal acidification process of M. tuberculosis. As shown in Fig. 3F and G, MCVs could recruit GFP-mCherry-LC3 molecules. The colocalization of M. tuberculosis with GFP-mCherry^+^ signal was decreased over time in both GML-bEnd.3 and GML-Raw 264.7 cells, suggesting that M. tuberculosis were degraded in acidified auto-lysosomes. Interestingly, the colocalization of M. tuberculosis with GFP^+^mCherry^+^ signal was retained at a low level over time in GML-bEnd.3 cells, whereas it was significantly increased in GML-Raw 264.7 cells (Fig. 3F and G), implying that M. tuberculosis could induce stronger inhibition of auto-lysosome acidification in GML-Raw 264.7 cells than in GML-bEnd.3 cells.

### Distinct transcriptional profile in different types of cells.

**FIG 4:**
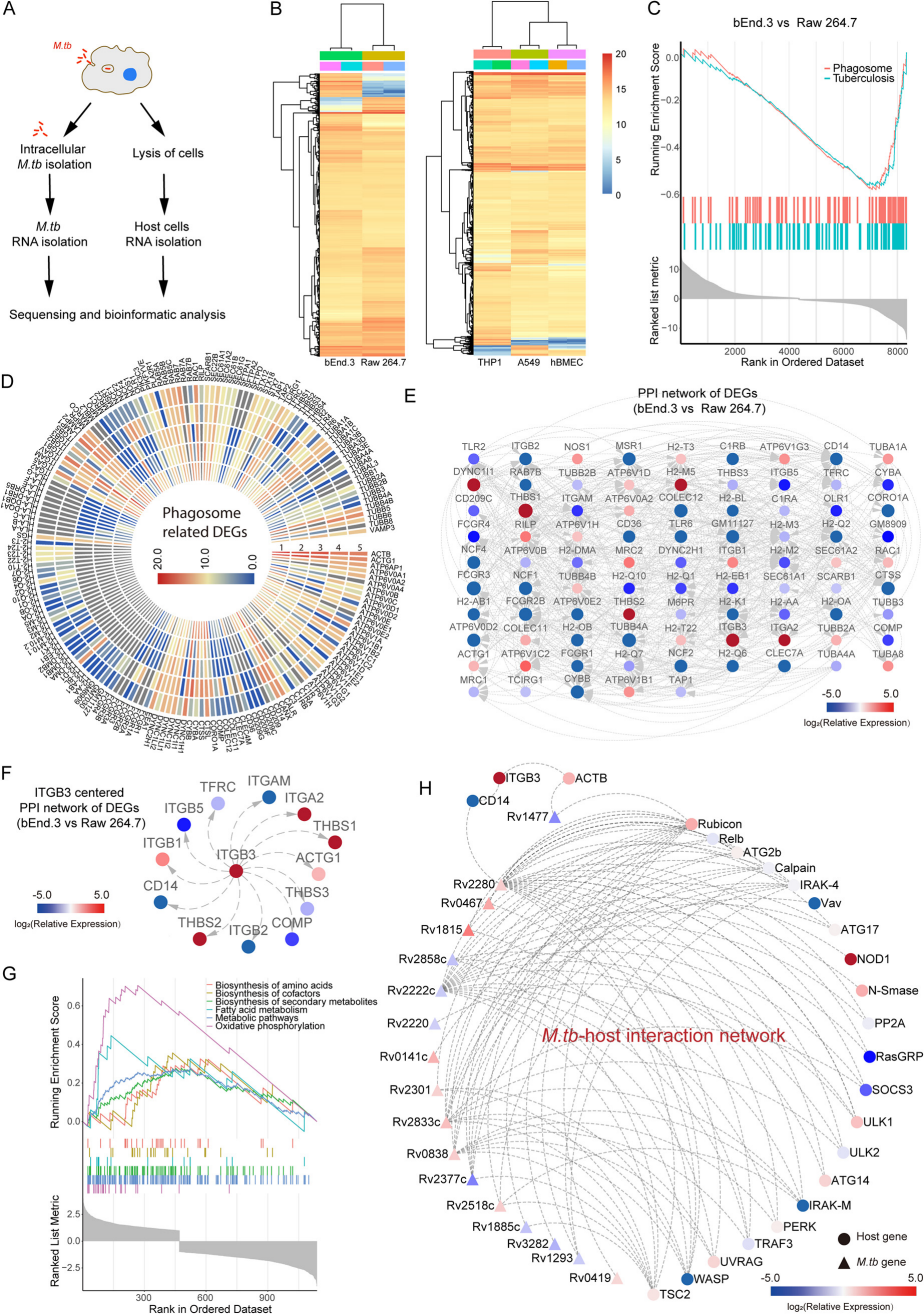
The distinct transcriptional profile of examined cell lines and intracellular M. tuberculosis. (A) Flowchart of transcriptional analysis. (B) Polarized heatmap showing the basal expression level of genes in bEnd.3, hBMEC, Raw 264.7, THP1, and A549 cells. (C) GSEA-KEGG analysis of DEGs between bEnd.3 and Raw 264.7 cells. (D) Basal expression of DEGs related to the phagosome pathway in uninfected cells (1, THP1; 2, A549; 3, hBMEC; 4, Raw 264.7; and 5, bEnd.3). (E) Protein-protein-interaction (PPI) network of phagosome-related DEGs between bEnd.3 and Raw 264.7 cells. (F) The *itgb3* PPI network extracted from D. (G) GSEA-KEGG enrichment of DEGs of intracellular M. tuberculosis isolated from bEnd.3 and Raw 264.7 cells. (H) The interaction network between M. tuberculosis DEG proteins and host proteins. Experiments were performed in duplicate.

As the distinct basal gene expression level and the M. tuberculosis infection response transcriptional profiles may contribute to different phagocytosis and autocytosis phenotypes in different cell lines, we then explored the transcriptional profiles of different host cells and the intracellular M. tuberculosis (Fig. 4A). The basal differentially expressed genes (DEGs) (*P*_adj_ < 0.05; abs [fold expression] > 2) of examined cells are shown in Fig. 4B The GSEA-KEGG analysis showed that the basal DEGs were mainly enriched in pathways related to phagosome activity, lysosome activity, platelet activation, and tuberculosis (Fig. 4C and Fig. S4A). Next, we analyzed the gene expression profiles of host cells in response to M. tuberculosis infection and found that the DEGs were enriched in phagosomes in bEnd.3 cells at day 3 postinfection, whereas this enrichment was not observed in other examined cells (Fig. S5A to G, Fig. S6A to O).

Considering the distinct trafficking processes of M. tuberculosis in examined cells, we compared the expression of phagosome-, lysosome-, and autophagy-related genes in different cells (Fig. 4D, Fig. S4B and C), and validated the expression of several genes by quantitative reverse transcriptase PCR (qRT-PCR) (Fig. S7). The protein-protein interactions (PPIs) network of DEGs (bEnd.3 versus Raw 264.7) was then constructed based on previous studies to further understand the function of DEGs (19) (Fig. 4E, Fig. S4D). By screening the potential genes involved in regulating M. tuberculosis trafficking in different host cells, we observed that the phagosome-related gene *itgb3* and the autophagy-related gene *atg3* were highly expressed in bEnd.3 cells (Fig. S4E, Fig. S7). As *itgb3* is highly expressed in bEnd.3 cells, we then extracted the PPI partners of *itgb3* from the DEG PPI network (Fig. 4F, Fig. S4F). Of note, the expression of most *itgb3*-interacting genes showed a significant difference between macrophages and endothelial cells (Fig. 4F, Fig. S4F). These data suggested that *itgb3* might be involved in regulating M. tuberculosis phagosomal trafficking and contributing to the distinct persistence and replication fate of M. tuberculosis in different cells.

Upon invading host cells, M. tuberculosis needs to adapt to the intracellular hostile environment by reprogramming its gene expression profile. Thus, to explore the different gene expression profiles of intracellular M. tuberculosis in macrophages and endothelial cells, we isolated intracellular M. tuberculosis from Raw 264.7 and bEnd.3 cells, respectively, transcriptome sequencing (RNA-seq). As shown in Fig. S4G, significant divergence over the transcriptome level could be observed between the intracellular M. tuberculosis isolated from bEnd.3 and Raw 264.7 cells. According to the GSEA-KEGG analysis, we found the DEGs were enriched for fatty acid metabolism, biosynthesis of amino acids, oxidative phosphorylation, etc. (Fig. 4G). The DEGs involved in such pathways were significantly more highly expressed in M. tuberculosis isolated from Raw 264.7 cells than from bEnd.3 cells (Fig. S4H and I), suggesting these DEGs might be involved in the adaption to the hostile environment of Raw 264.7 cells.

As M. tuberculosis secreted proteins and cell wall proteins may interfere with host immune response to aid the bacterium’s long-term survival, we constructed an M. tuberculosis-host PPI network between 17 M. tuberculosis DEGs and host proteins according to the previously established global M. tuberculosis-host PPI network (Fig. 4H). Among these genes, Rv1477, a secreted peptidoglycan hydrolase, was found to interact with ACTB, which is an interaction partner of ITGB3 (20). Meanwhile, we observed that Rv2280 interacted with CD14, which is also an interaction partner of ITGB3. It could be possible that these ITGB3-ACTB-Rv1477 and ITGB3-CD14-Rv2280 axes were involved in regulating the survival of M. tuberculosis in host cells, of which the underlying mechanisms needed to be further investigated.

### Knockdown of *itgb3* and *atg3* enhanced M. tuberculosis survival in bEnd.3 cells.

**FIG 5:**
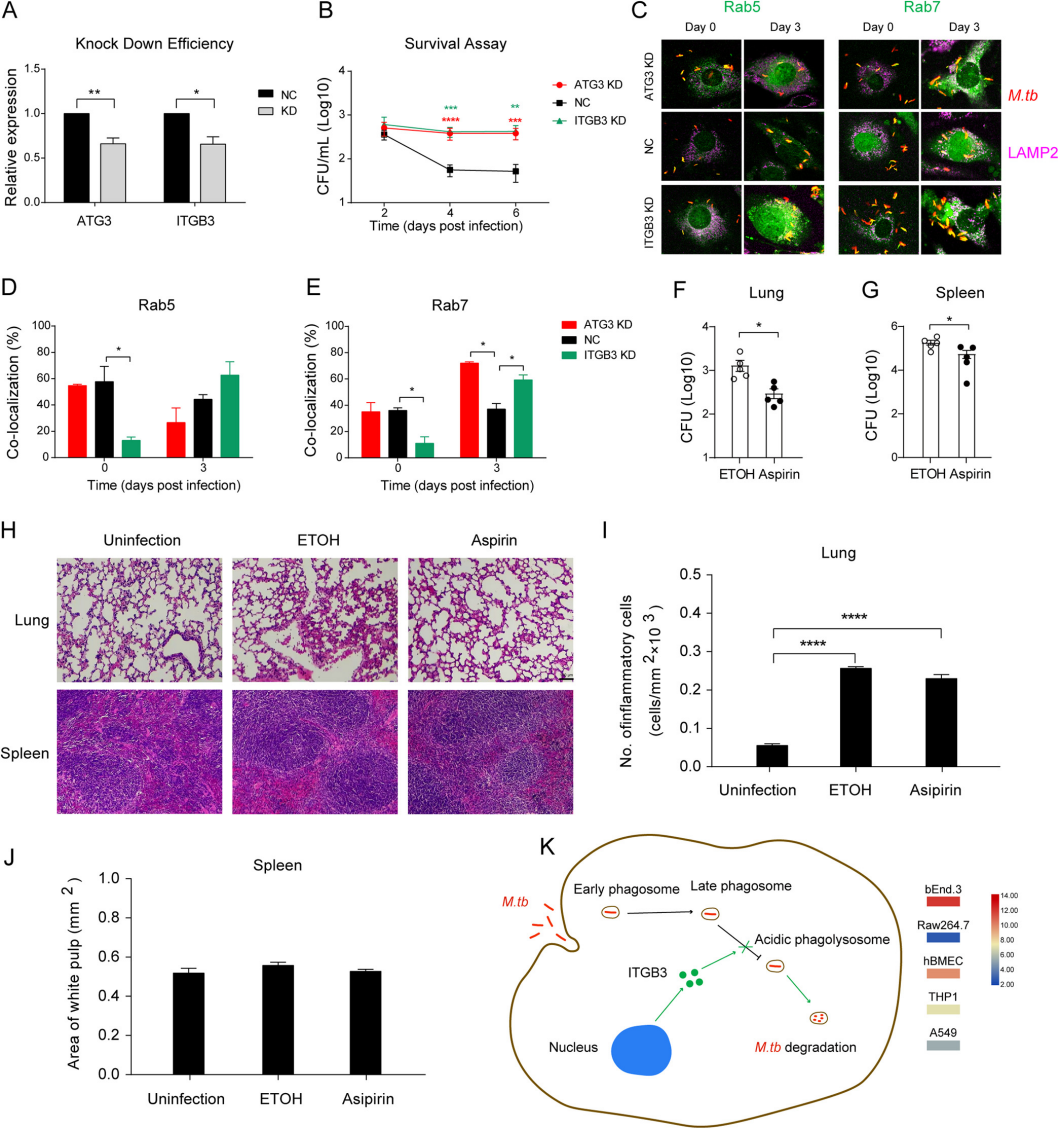
ITGB3 and ATG3 facilitate M. tuberculosis survival by interfering with phagosomal trafficking. (A) Knockdown efficiency of ITGB3 and ATG3 in bEnd.3 cells. (B) Survival assay. Cells were seeded in a 24-well plate and infected with M. tuberculosis for 6 h and then lysed at the indicated time points for viable intracellular counting. (C) Confocal microscopy images as indicated. Cells were seeded onto coverslips and infected with dsRed-expressing M. tuberculosis for 6 h. Cells were fixed at the indicated time points and incubated with rabbit anti-Rab5, rabbit anti-Rab7, and rat anti-LAMP2 antibodies, respectively. Alexa Fluor 405 goat anti-rabbit IgG (green) was used to detect Rab5 and Rab7. Alexa Fluor 647 goat anti-rat IgG (purple) was used to detect LAMP2. All specimens were imaged with the confocal microscope. (D and E) The colocalization of M. tuberculosis with Rab5 (D) and Rab7 (E) was quantified. (F and G) Mycobacterial load in the lung and spleen. Mice were infected with M. tuberculosis and then administered with aspirin for 6 days. Lungs and spleens were obtained on day 7 and homogenized for CFU. (H) The H&E staining of lungs and spleens as indicated. Scale bars = 100 μm. (I) The histopathology of lungs and spleens at 7 days postinfection was analyzed with ImageJ and quantified by analyzing the number of inflammatory cells. (J) The histopathology of spleens was analyzed with ImageJ and quantified by analyzing the area of white pulps. (K) Schematic of how ITGB3 may overwhelm phagosomal maturation arrest and cause M. tuberculosis degradation in host cells. The expression of *itgb3* in all examined cell lines is shown on the side. Experiments were performed in triplicate and repeated at least three times. Animal experiments were repeated two times with 5 to 7 mice per group each time. Data are presented as mean ± SEM and analyzed by unpaired *t* test with two-tailed *P* values. The values of *, *P < *0.05; **, *P < *0.01; ***, *P < *0.001; and ****, *P < *0.0001 were considered statistically significant differences.

As the phagosome-related gene *itgb3* and the autophagy-related gene *atg3* showed the highest basal expression levels in bEnd.3 cells, we hypothesized that these genes might be involved in bacteria elimination and replication inhibition by interfering with trafficking pathways. To test this, we constructed *itgb3* and *atg3* knockdown bEnd.3 cell lines (Fig. 5A, Fig. S8A) and investigated the effect of knocked down *itgb3* or *atg3* on M. tuberculosis intracellular survival. Our data showed that upon *atg3* or *itgb3* knockdown, the intracellular survival of M. tuberculosis was significantly increased compared to the control (Fig. 5B). Furthermore, the colocalization of M. tuberculosis with Rab5 was increased upon *itgb3* knockdown in bEnd.3 cells at day 3 postinfection, while the colocalization of M. tuberculosis with Rab5 was decreased upon *atg3* knockdown at day 3 postinfection (Fig. 5C and D). Notably, the colocalization of M. tuberculosis with Rab7 was significantly increased upon *itgb3* and *atg3* knockdown in bEnd.3 cells (Fig. 5C and E). The colocalizations of M. tuberculosis with Rab7, LAMP2, cathepsin L, LAMP2, and LC3 were also investigated and showed no significant differences between the *itgb3* and *atg3* knockdown cells and the control cells at day 3 postinfection (Fig. S8B to F).

### ITGB3 agonist treatment facilitated M. tuberculosis clearance *in vivo*.

As ITGB3 may function as a potential host restriction factor for limiting M. tuberculosis replication, we further examined the role of ITGB3 in controlling M. tuberculosis survival *in vivo* through the treatment of mice with aspirin, an ITGB3 agonist (21). Upon treatment with aspirin, the bacterial load in the lung and spleen was significantly decreased compared to ethanol (ETOH)-treated control mice on day 7 postinfection (Fig. 5F and G). However, no significant difference was observed between ETOH-treated mice and aspirin-treated mice on day 21 postinfection (Fig. S9A and B).

Hematoxylin and eosin (H&E) staining showed that the alveolar wall was incomplete or thickened in the ETOH group. Of note, the aspirin treatment could recover the alveolar wall morphology on day 7 postinfection, but not on day 21 postinfection (Fig. 5H, Fig. S9C), and there was no significant difference in the spleen between aspirin-treated groups and control groups (Fig. 5H to J, Fig. S9C and E). Furthermore, the histopathology quantification of inflammation showed that the aspirin treatment could reduce inflammation in the lung of mice on day 7 postinfection but not on day 21 postinfection (Fig. 5I, Fig. S9C and D). The possible reason might be that aspirin exerts its function mainly during the innate immunoresponse stage. Together, our data suggested that high expression of ITGB3 might facilitate bEnd.3 cells to eliminate invading M. tuberculosis by overwhelming phagolysosome maturation arrest and activation of ITGB3 could facilitate M. tuberculosis clearance *in vivo* (Fig. 5H).

## DISCUSSION

As an aerosol transmission pathogen, M. tuberculosis is engulfed by macrophages, epithelial cells, and endothelial cells (7, 8, 22). Upon internalization into host cells, M. tuberculosis undergoes distinct trafficking processes in different cells. We observed M. tuberculosis strain H37Ra could arrest phagosome maturation at early stages in macrophages and late stages in endothelial and epithelial cells. M. tuberculosis H37Ra was degraded at a different level in acidified lysosomes (37.3% in THP1, 41% in Raw 264.7, 42% in hBMEC, and 19.6% in A549 cells). For strain H37Rv, the vacuoles maintained a low level of colocalization with phagosome markers over time in both THP1 and hBMEC cells. Notably, 86.3% of M. tuberculosis H37Ra was trafficked into the acidified lysosome for degradation in bEnd.3 cells, implying that the decreased numbers of viable M. tuberculosis in bEnd.3 cells might be caused by this acidified-lysosome-mediated degradation, although the M. tuberculosis could partially arrest late phagosome fusion with a lysosome in some unknown ways. These data suggest that different cells are equipped with different defense mechanisms to eliminate invading bacteria.

Autophagy is involved in innate defense against invading pathogens by sequestering them into autophagosomes and delivering them to lysosomes. We showed that M. tuberculosis could inhibit autophagosome formation in THP1 and hBMEC cells, but were delivered into auto-lysosomes for degradation in A549, Raw 264.7, and bEnd.3 cells. Our data demonstrate that M. tuberculosis can be degraded in acidified auto-lysosomes in bEnd.3 cells, but not in Raw 264.7 cells. By analyzing autophagy-related genes in different cells, we found that bEnd.3 cells highly expressed the *atg3* gene. The E2 enzyme ATG3 conjugates the ubiquitin-like protein ATG8 to phosphatidylethanolamine to drive autophagosome formation (23). Upon *atg3* knockdown, we observed that the viable intracellular M. tuberculosis was significantly increased and the phagosome maturation was inhibited at late stages. As bEnd.3 is a brain microvascular endothelial cell line often used as a blood-brain barrier model, it would be conceivable that this type of cell might be equipped with more robust immunologic machinery for brain pathogen defense.

Further, we analyzed the genes involved in the phagocytosis pathway and found that the basal expression of ITGB3 is significantly higher in bEnd.3 cells in comparison to other examined cells. Importantly, when ITGB3 was knocked down, M. tuberculosis survived better in bEnd.3 cells than in wild-type cells. Upon ITGB3 knockdown, the colocalization of MCVs with Rab7 was significantly enhanced, suggesting that less M. tuberculosis was delivered into lysosomes for degradation. Thus, we hypothesize that ITGB3 may facilitate M. tuberculosis clearance by delivering bacilli to phagolysosomes. Notably, in a cohort of TB patients, significant alteration of *itgb3* mRNA was observed (24). Moreover, it has been reported that treatment with acetylsalicylic acid (aspirin), an ITGB3 agonist, in zebrafish could markedly reduce mycobacteria burden (21). Consistent with this, we found that aspirin treatment could attenuate M. tuberculosis survival in mice, indicating that ITGB3 might be a potential target for anti-M. tuberculosis drug development.

M. tuberculosis has a characteristic lipid-rich cell wall to counter host intracellular hostile events and allow its proliferation in a niche. The gene expression profiles of intracellular M. tuberculosis isolated from endothelial cells and macrophages showed that DEGs highly expressed in intracellular *M. tuberculosis* isolated from macrophages were enriched in fatty acid metabolism pathways. These data suggest that M. tuberculosis may upregulate its lipid synthesis-related gene expression to synthesize more lipids to counteract the hostile environments in host cells. Moreover, the expression of ATP synthase and NADH-quinone oxidoreductase genes were significantly lower in intracellular M. tuberculosis of bEnd.3 cells, which may reflect a reduced metabolic level and restricted life cycle of M. tuberculosis in these cells. Notably, we found that 17 DEGs interacted with host genes, of which Rv1477 (RipA) showed significantly lower expression in endothelial cells than in macrophages. Rv1477 is essential for M. tuberculosis cell wall synthesis and division and the depletion of Rv1477 could promote the clearance of M. tuberculosis in infected mice (25). As Rv1477 formed a complex with ITGB3 through ACTB, it would be important to further investigate whether Rv1477 may participate in ITGB3-mediated phagosomal maturation arrest.

Together, our data demonstrated that different cells are equipped with different levels of pathogen-host restriction factors, such as ITGB3 and ATG3, to eliminate pathogen invasion. M. tuberculosis could significantly inhibit lysosome acidification to promote replication or long-term persistence in THP1, hBMEC, A549, and Raw 264.7 cells, whereas M. tuberculosis is unable to replicate in bEnd.3 cells because the host cell can deliver them into acidified phagolysosomes and auto-lysosomes. Our data identified that ITGB3 and ATG3 may function as host restriction factors to eliminate M. tuberculosis in bEnd.3 cells. These findings may shed a light on the understanding of the innate immunity against M. tuberculosis invasion and facilitate the identification of anti-M. tuberculosis factors for drug development and transgenetic breeding of M. tuberculosis-resistant animals.

## MATERIALS AND METHODS

### Bacteria and cell cultures.

M. tuberculosis H37Ra was cultured in Middlebrook 7H9 broth (Becton, Dickinson) supplemented with 0.5% glycerol, 0.05% Tween 80, and 10% oleic acid albumin dextrose catalase (OADC, Becton, Dickinson). For confocal microscopy, M. tuberculosis cells were transformed with a plasmid expressing dsRed or blue fluorescent protein (BFP). For infection, the bacterial culture optical densities at 600 nm (OD_600_) were adjusted to achieve the required multiplicity of infection (MOI) and centrifuged at 3,000 × *g* for 10 min to pellet the bacteria. The pellet was resuspended in an infection medium and passed through an insulin syringe to disperse the bacteria. In addition, 50 μl aliquots of serially diluted cultures were plated onto Middlebrook 7H11 agar (Becton, Dickinson) to determine the number of viable bacteria (CFU).

Murine brain microvascular endothelial cells (bEnd.3, ATCC CRL-2299), murine macrophages (Raw 264.7, ATCC TIB-71TM), and HEK293T cells were maintained in Dulbecco’s modified Eagle medium (DMEM) supplemented with 10% fetal bovine serum (FBS). Human lung epithelial cells (A549, ATCC CCL-185) were maintained in F-12K medium supplemented with 10% FBS. Human monocytes (THP1, ATCC TIB-202) were maintained in RPMI 1640 medium supplemented with 10% FBS and were differentiated for 24 h using a culture medium containing 40 ng/ml phorbol 12-myristate 13-acetate (PMA) before infection. Human brain microvascular endothelial cells (hBMECs) were maintained in RPMI 1640 medium supplemented with 10% FBS, 2 mM l-glutamine, 1 mM sodium pyruvate, MEM nonessential amino acids, MEM amino acids, and MEM vitamins. To construct GFPph-mCherry-LC3 (GML) stable cell lines, bEnd.3 and Raw 264.7 cells were transfected with pX335-gRNA and GFPph-mCherry-LC3 and purified by fluorescence-activated cell sorting (FACS) (BD, CA, USA), as previously described (19).

### Construction of gene knockdown cell lines.

Short hairpin RNAs (shRNAs) targeting *atg3* or *itgb3* were designed using BLOCK-iT RNAi Designer (Invitrogen) (shown in Table 1) and inserted into the pHKO vector. To obtain lentiviruses, HEK293T cells were seeded into 6-well plates and cotransfected with 1 μg pMD.2G, 2 μg pSPAX, and 2 μg pHKO-*atg3*-kd-shRNAs or pHKO-*itgb3*-kd-shRNAs. pHKO vector alone was used as the negative control. At 48 h posttransfection, the supernatants were collected and filtered with a 0.45-μm filter. Viruses were pelleted by centrifuging 30 min at 5,000 × *g* at 4°C after overnight incubation with virus concentration solution. Viral pellets were resuspended, mixed with polybrene, and added to bEnd.3 cells. After 48 h, the medium was replaced with fresh medium containing 10% FBS and 4 μg/ml puromycin (s7417, Selleck) to purify the cell lines.

**TABLE 1:** Oligonucleotides used in this study

Name	Sequence
bEnd.3-ATG3-shRNA-F	CCGGGCTTGGTGTTCATATGTATCTTTCAAGAGAAGATACATATGAACACCAAGCTTTTTTGGTACC
bEnd.3-ATG3-shRNA-R	AATTGGTACCAAAAAAGCTTGGTGTTCATATGTATCTTCTCTTGAAAGATACATATGAACACCAAGC
bEnd.3-ITGB3-shRNA-F	CCGGGCTGATGACTGAGAAACTATCTTCAAGAGAGATAGTTTCTCAGTCATCAGCTTTTTTGGTACC
bEnd.3-ITGB3-shRNA-R	AATTGGTACCAAAAAAGCTGATGACTGAGAAACTATCTCTCTTGAAGATAGTTTCTCAGTCATCAGC
mATG3-qPCR-F	TGACGCTGGAGGTGAAGATGC
mATG3-qPCR-R	AAGGCTGCCGTTGCTCATCAT
mCOLEC12-qPCR-F	CACCTGGTCCAACTGGCAACA
mCOLEC12-qPCR-R	TTGGGACCCTGTGAGCCTTTG
mCORO1A-qPCR-F	TCTGATCTGTGAGGCCAGTG
mCORO1A-qPCR-R	CCTCAGAGCCACTGGCAATG
mIDS-qPCR-F	TGTTGCTAGGCTCCTTCTGC
mIDS-qPCR-R	ACGCTATGGGATGCCAGCTG
mITGB3-qPCR-F	ATCCTGGTGGTCCTGCTGTCA
mITGB3-qPCR-R	TGGCTCTGGCTCGTTCTTCCT
mGAPDH-F	AGGTCGGTGTGAACGGATTTGG
mGAPDH-R	CGTTGAATTTGCCGTGAGTGGA
hATG3-qPCR-F	TCCCATGTGTTCAGTTCACCC
hATG3-qPCR-R	TGCCACTAATCTTACATACAGGG
hCOLEC12-qPCR-F	AGGGCAATCTGCAGAACCAA
hCOLEC12-qPCR-R	TGAAAGTCGTTCTTGATTCG
hCORO1A-qPCR-F	GGCTTTTGGGGGATCACTGT
hCORO1A-qPCR-R	TGTGAAGAGCCCCAGTCTTG
hIDS-qPCR-F	ACCGATGATTCTCCGTATAG
hIDS-qPCR-R	CTCAGTGCTCTGTTTGTCAG
hITGB3-qPCR-F	TTGGAGACACGGTGAGCTTC
hITGB3-qPCR-R	TTAGGTTCAGCTTGGGCCTG
hGAPDH-F	GTCTCCTCTGACTTCAACAGCG
hGAPDH-R	ACCACCCTGTTGCTGTAGCCAA
Rv3722c-qPCR-F	CATCGCAGTGCCCAACCTGAT
Rv3722c-qPCR-R	ATGCCGTCCTGCTCCTGAATC
Rv2709-qPCR-F	AATGGCCAGTTCGACGACTT
Rv2709-qPCR-R	AGATCAGTCCGTTGTGCCAG
Rv2801c-qPCR-F	AGGTAGCGAAGCGAACAACC
Rv2801c-qPCR-R	ACGGATAGACCTTGGCGATG
Rv3444c-qPCR-F	CGAATACTCCGTTCGTCAGGA
Rv3444c-qPCR-R	TTGAGTTGCTCCGCGTGGTA
Rv0847-qPCR-F	GGTCGCGCTGGGGGTGACG
Rv0847-qPCR-R	CCAGGTGCCCGTGGTCGGT
gyrB-qPCR-F	AAGGACCGCAAGCTACTGAA
gyrB-qPCR-R	CAGACCTTCTGCACAAACGA

Quantitative reverse transcriptase PCR (qRT-PCR) was performed to detect the knockdown efficiency of target genes. RNAs (1 μg) were reverse transcribed into cDNA using ReverTra Ace qPCR RT Master Mix (Toyobo). Gene expression was analyzed by qRT-PCR using SYBR green Master Mix (Invitrogen) on the ABI 7300 system (Applied Biosystems). Data were analyzed using the threshold cycle (2^-ΔΔCt^) method relative to GAPDH levels for each sample. The primers used for qRT-PCR are shown in Table 1.

### Intracellular bacterial viability assay.

Cells (5.0 × 10^4^) were seeded into a 24-well plate for 24 h and were incubated with M. tuberculosis (MOI = 10) for 6 h at 37°C in a 5% CO_2_ incubator. The cells were washed three times with prewarmed phosphate-buffered saline PBS to remove extracellular M. tuberculosis and supplied with a fresh medium with 5% FBS containing amikacin (50 μg/ml) (referred to as day 0). The medium was changed every 2 days to avoid serum starvation-induced autophagy. The infected cells were lysed at the indicated time points using sterile 0.1% Tween 80 in water, and viable M. tuberculosis were enumerated by serial dilution of lysates and plating as described above.

### Cell proliferation analysis.

Cells (5.0 × 10^4^) were seeded onto coverslips in a 24-well plate for 24 h and were incubated with dsRed expressing M. tuberculosis (MOI = 10) for 6 h at 37°C with 5% CO_2_. The cells were washed three times with prewarmed PBS to remove extracellular M. tuberculosis and supplied with fresh medium with 5% FBS containing amikacin (50 μg/ml). At the indicated time points, cells were detached with 0.25% (wt/vol) trypsin-0.03% (wt/vol) EDTA solution (Gibco) for 5 min at 37°C. Live cells were counted after staining with 0.4% trypan blue solution.

### Confocal microscopy.

For fluorescence visualization, 5.0 × 10^4^ cells were seeded onto coverslips in a 24-well plate for 24 h and infected with dsRed gene-expressing M. tuberculosis (MOI = 10) for 6 h at 37°C with 5% CO_2_. The infected cells were fixed with 4% paraformaldehyde overnight at the indicated time points. Specimens were incubated with DAPI (4′,6-diamidino-2-phenylindol) (1:5,000, Invitrogen) for 5 min and then mounted onto microscope slides using Prolong antifade reagent (Invitrogen).

For trafficking assays, the infected cells were fixed at the indicated time points with 4% paraformaldehyde. After blocking with 5% normal goat serum in 0.3% Triton X-100 PBS for 1 h at room temperature, specimens were incubated with a 1:300 dilution of anti-Rab5 (Abcam), anti-Rab7 (Santa Cruz Biotechnology), anti-LAMP2 (Abcam), anti-cathepsin L (Abcam) or anti-LC3 (Sigma) antibody for 2 h at room temperature. Alexa Fluor 488 goat anti-rabbit IgG (Invitrogen) or Alexa Fluor 405 goat anti-rabbit IgG was used with a 1:300 dilution to detect Rab5, Rab7, cathepsin L, and LC3. Alexa Fluor 647 goat anti-rat IgG (Invitrogen) was used with a 1:300 dilution to detect LAMP2. The specimens were incubated with DAPI in PBS for 5 min. After washing 3 times with PBS, the specimens were mounted onto microscope slides using Prolong antifade reagent.

For auto-lysosomal acidification analysis, 5.0 × 10^4^ GML-bEnd.3 and GML-Raw 264.7 cells were seeded onto coverslips in a 24-well plate for 24 h and infected with BFP-expressing M. tuberculosis (MOI = 10) for 6 h at 37°C in 5% CO_2_. The infected cells were fixed with 4% paraformaldehyde overnight at the indicated time points and then mounted onto microscope slides using Prolong antifade reagent.

Images were obtained with an Olympus confocal laser microscope system equipped with FV10 ASW Imaging Software (Version 4.2; Olympus. Japan). One hundred bacteria in total were analyzed for each assay. All experiments were performed in triplicate.

### Western blotting.

Cells (1.0 × 10^6^) were seeded into a 6-well plate and then infected with M. tuberculosis (MOI = 10) for 6 h at 37°C in 5% CO_2_. The cells were lysed with radioimmunoprecipitation assay (RIPA) lysis buffer at the indicated time points. The cell lysates mixed with loading buffer were boiled for 10 min and cooled on ice for 10 min. The proteins were separated by 12.5% SDS-PAGE gels and then transferred to polyvinylidene difluoride (PVDF) membranes. The membrane was blocked with 5% (m/v) skim milk in Tris-buffered saline with Tween 20 (TBST) buffer (50 mM Tris, pH 7.5, 150 mM NaCl, 0.05% Tween 20) at room temperature for 1 h and incubated with primary antibodies against LC3, Beclin, ITGB3, or ATG*3* (Abclonal) overnight at 4°C. The membrane was washed three times with TBS-0.05% Tween 20 and then incubated with horseradish peroxidase (HRP)-conjugated goat anti-rabbit or anti-mouse secondary antibody (1:5,000, ABclonal) for 1 h. The specific proteins were visualized using an electro-chemiluminescence ECL kit under Image Lab software 4.0.1 (Bio-Rad).

### Host cells RNA-seq library preparation and sequencing.

Cells (1.0 × 10^6^) were seeded into a 6-well plate and then infected with M. tuberculosis (MOI = 10) for 6 h at 37°C in 5% CO_2_. The cells were lysed with 1 ml of TRIzol reagent at the indicated time points. Lysates were mixed with 0.2 ml of chloroform centrifuge for 10 min at 12,000 × *g* at 4°C. Then, 0.4 ml of the aqueous phase was mixed with 0.4 ml of isopropanol in a new nuclease-free Eppendorf tube. The RNA was precipitated at room temperature for 10 min and pelleted by centrifugation for 10 min at 12,000 × *g*. Eluted RNAs were washed twice with 75% ethanol (ETOH). The quality and quantity of RNAs were examined by Nanodrop 2000c spectrophotometer (Thermo Fisher Scientific). DNAs were removed using Ambion TURBO DNA-free DNase treatment and removal reagents (Invitrogen) following the user guide.

The libraries were generated with VAHTS Stranded mRNA-seq library prep kit (Vazyme Biotech Co., Ltd, China). Briefly, mRNAs were isolated using mRNA capture beads from 1 μg of total RNA and incubated for fragmentation at 94°C for 8 min to get mRNAs with a length of around 150 bp to 200 bp. The mRNAs were reverse transcribed to cDNA and converted into double-stranded cDNA molecules. Following end repair and dA tailing, the paired-end sequencing adaptors were ligated to the ends of the cDNA fragments and then subjected to library amplification and purification. The purified libraries were validated and quantified using the Agilent 2100 Bioanalyzer (Agilent Technologies) and sequenced with the Hi-Seq 10× instrument (Illumina).

### Intracellular M. tuberculosis RNA-seq library preparation and sequencing.

Raw 264.7 and bEnd.3 cells were seeded into T225 flasks and infected with M. tuberculosis (MOI = 10) for 6 h at 37°C in 5% CO_2_. Infected cells were lysed with 30 ml of guanidine thiocyanate (GTC) buffer at day 3 postinfection and centrifuged for 20 min at 5,000 × *g* to pellet bacteria. The bacteria pellets were suspended in 1 ml of GTC and centrifuged for 2 min at 15,000 × *g*. The bacteria pellets were disturbed in 1 ml of TRIzol reagent by vortex for 10 min. Total RNAs were extracted as described above. Ribosomal RNA (rRNA) was removed under the guidelines of Ribo-Zero rRNA removal kit (Illumina). Library construction and sequencing were performed as described above.

### Data analysis pipeline.

The Hisat2 index required for human and murine cells RNA-seq data alignment was downloaded from ftp://ftp.ccb.jhu.edu/pub/infphilo/hisat2/data. The M. tuberculosis hisat2 index was constructed by genome sequence and annotation file available on ftp://ftp.ensemblgenomes.org/pub/bacteria/release-51/fasta/bacteria_5_collection/mycobacterium_tuberculosis_h37ra_gca_000016145/dna/Mycobacterium_tuberculosis_h37ra_gca_000016145.ASM1614v1.dna.toplevel.fa.gz and ftp://ftp.ensemblgenomes.org/pub/bacteria/release-51/gtf/bacteria_5_collection/mycobacterium_tuberculosis_h37ra_gca_000016145/Mycobacterium_tuberculosis_h37ra_gca_000016145.ASM1614v1.51.gtf.gz, respectively. The alignment was completed using the protocol provided by Pertea et al. (26). The mapping result was counted with the Htseq (27).

For the differential analysis, all of the gene expression count tables were merged into a combined matrix showing the expression count of each gene from every individual sample. DEseq2 was used to export the foldchange list and normalized count matrix. The gene ID in the matrix was annotated with DAVID (https://david.ncifcrf.gov/).

The expression levels of autophagy, phagosome, and lysosome pathway genes were normalized corresponding to each sample. GSEA-KEGG analysis and KEGG enrichment were performed by R package clusterProfiler (28) and visualized by R package enrichplot (https://github.com/GuangchuangYu/enrichplot). The polarized heatmap was generated by TBtools with the cells filling indicating the expression level. The interaction network was constructed by Cytoscape (29) with the host protein interaction based on string-db (20) and M. tuberculosis-host PPI based on our previous study (19). The bar plotted heat map was generated by R package pheatmap (https://CRAN.R-project.org/package=pheatmap). Differential expression genes (DEG) between cell lines were selected with the following principle: Abs (log_2_foldchange [gene expression in A cell comparing to uninfected A cell] −log_2_change [gene expression in B cell comparing to uninfected B cell]) > 1 or gene expression cannot be detected in A or B cells, but abs (log_2_foldchange [gene expression in B or A cell comparing to uninfected B or A cell]) > 1.

### Animal experiment.

The animal experiment was carried out under the administrations of animal care and usage for research of the Ministry of Health in China. Animals were raised in specific-pathogen-free condition rooms. All the experiments were approved by the committee on the ethics of animal experiments of the College of Veterinary Medicine, Huazhong Agricultural University (HZAUMO-2020-0004).

C57BL/6 mice (6-week-old, female, 20 ± 2 g) were purchased from the Laboratory Animal Center of Huazhong Agricultural University. All animals were randomly distributed into 3 groups, 5 to 7 of each. Mice were infected intravenously with M. tuberculosis at a dose of 5 × 10^6^ CFU. Then, mice received oral aspirin (ASA) at a daily dose of 10 mg/kg (30). The ETOH intake group was used as an untreated control and PBS was used as an uninfected control. These mice were administered with drugs for 6 days or 20 days and euthanized at day 7 or 21 postinfection, respectively. Lungs and spleens were used for histopathological observation and CFU analysis. A part of the left lung and spleen were fixed with 4% paraformaldehyde for 48 h, and tissues were paraffin embedded to make a section for hematoxylin and eosin (H&E) staining. Homogenates of lungs were plated onto Middlebrook 7H11 agar containing OADC enrichment and BBL MGIT PENTA antibiotics (Becton, Dickinson) for bacterial burden enumeration. CFU were counted after incubation at 37°C for 3 to 4 weeks. Experiments were repeated two times.

### Statistical analysis.

Data were analyzed and plotted using GraphPad Prism 6.0 (La Jolla, CA). Data are expressed as mean ± standard error of the mean (SEM). Evaluation of the significance of differences between groups was performed by unpaired *t* test as indicated.

### Data availability.

The data sets and computer code produced in this study are available in the following databases: Host RNA-Seq data: Gene Expression Omnibus GSE125907 and M. tuberculosis RNA-Seq data: Gene Expression Omnibus GSE162200.

10.1128/mSystems.00783-21.3 FIG S3 Western blotting. (A) Detection of LC3 and Beclin-1 in endothelial cells, macrophages, and epithelial cells. Cells were seeded in a 6-well plate and infected with M. tuberculosis H37Ra for 6 h and then lysed with RIPA lysis buffer at the indicated time points. The LC3 and Beclin-1 proteins were detected by rabbit anti-LC3 and rabbit anti-beclin antibodies, respectively. All experiments were performed in triplicate. The quantity of each band was analyzed using Image J. Download FIG S3, PDF file, 1.3 MB. Copyright © 2021 Chen et al. 2021 Chen et al. https://creativecommons.org/licenses/by/4.0/ This content is distributed under the terms of the Creative Commons Attribution 4.0 International license.

10.1128/mSystems.00783-21.4 FIG S4 Transcriptional profiles of examined cell lines. (A) GSEA-KEGG analysis of gene expression between hBMEC and THP1 cells. (B) Basal expression of DEGs related to the lysosome pathway in infected cells. (C) Basal expression of DEGs related to the autophagy pathway in infected cells. (1, THP1; 2, A549; 3, hBMECs; 4, Raw 264.7; 5, bEnd.3). (D) Protein-protein-interaction (PPI) network of phagosome-related DEGs between hBMEC and THP1 cells. (E) Volcano plot of DEGs between endothelial cells (bEnd.3 and hBMEC) and macrophages (Raw 264.7 and THP1). (F) ITGB3 PPI network extracted from D. (G) Gene expression profiles of intracellular M. tuberculosis isolated from Raw 264.7 and bEnd.3 cells. (H) The normalized gene expression levels of oxidative phosphorylation-related genes in intracellular M. tuberculosis in bEnd.3 cells and Raw 264.7 cells. (I) The normalized gene expression levels of fatty acid metabolism-related DEGs of intracellular M. tuberculosis from bEnd.3 cells and Raw 264.7 cells. Download FIG S4, PDF file, 7.2 MB. Copyright © 2021 Chen et al. 2021 Chen et al. https://creativecommons.org/licenses/by/4.0/ This content is distributed under the terms of the Creative Commons Attribution 4.0 International license.

10.1128/mSystems.00783-21.5 FIG S5 KEGG enrichment of DEGs after M. tuberculosis infection in each cell at different time points. Download FIG S5, PDF file, 0.9 MB. Copyright © 2021 Chen et al. 2021 Chen et al. https://creativecommons.org/licenses/by/4.0/ This content is distributed under the terms of the Creative Commons Attribution 4.0 International license.

10.1128/mSystems.00783-21.6 FIG S6 Gene profiles of different cell lines in response to M. tuberculosis invasion. (A and B) Heatmap of DEGs of bEnd.3 cells relative to Raw 264.7 cells at day 0 and day 3 postinfection. (C and D) Heatmap of DEGs of hBMECs relative to THP1 cells at day 0 and day 3 postinfection. (E and F) Heatmap of DEGs of A549 cells relative to THP1 cells at day 0 and day 3postinfection. (G and H) Heatmap of DEGs of hBMECs relative to A549 cells at day 0 and day 3 postinfection. (I and J) KEGG enrichment of DEGs of bEnd.3 cells relative to Raw 264.7 cells at day 0 and day 3 postinfection. (K and L) KEGG enrichment of DEGs of hBMECs relative to THP1 cells at day 0 and day 3 postinfection. (M and N) KEGG enrichment of DEGs of A549 cells relative to THP1 cells at day 0 and day 3 postinfection. (O) KEGG enrichment of DEGs of hBMECs relative to A549 cells at day 0. Download FIG S6, PDF file, 2.4 MB. Copyright © 2021 Chen et al. 2021 Chen et al. https://creativecommons.org/licenses/by/4.0/ This content is distributed under the terms of the Creative Commons Attribution 4.0 International license.

10.1128/mSystems.00783-21.7 FIG S7 qRT-PCR verification of RNA-seq data. (A) The basal expression of ATG3, COLEC12, CORO1A, IDS, and ITGB3 in endothelial cells, macrophages, and epithelial cells. Cells were seeded in a 6-well plate and then infected with M. tuberculosis for 6 h. Cells were lysed with Trizol reagent for RNA extraction. RNAs were then transcribed to cDNA for qRT-PCR. GAPDH was used as a control. Data are shown in Δ*C_T_*. (B) The relative expression of Rv3722c, Rv3444c, Rv0847, Rv2801, and Rv2709 from intracellular *M. tuberculosis* isolated from macrophages. Intracellular M. tuberculosis H37Ra was isolated from Raw 264.7 cells at day 3 postinfection. Bacterial RNAs were extracted using the Trizol method and then transcribed to cDNA for qRT-PCR; *gyrB* was used as a control. Data are shown in 2^-ΔΔCt^. All experiments were performed in triplicate. Data are presented as mean ± SEM and analyzed by unpaired *t* test with a two-tailed *P* value: ***, *P* < 0.001; ****, *P* < 0.0001. Download FIG S7, PDF file, 0.8 MB. Copyright © 2021 Chen et al. 2021 Chen et al. https://creativecommons.org/licenses/by/4.0/ This content is distributed under the terms of the Creative Commons Attribution 4.0 International license.

10.1128/mSystems.00783-21.8 FIG S8 Knockdown efficiency and trafficking analysis of M. tuberculosis upon ITGB3 and ATG3 knockdown in bEnd.3 cells. (A) Knockdown efficiency estimation by western blotting. Cells were seeded in a 6-well plate respectively for 24 h and then lysed with RIPA lysis buffer for western blotting. The ITGB3 and ATG3 proteins were detected by rabbit anti-ITGB3 and rabbit anti-ATG3 antibodies, respectively. For trafficking analysis, cells were seeded onto coverslips and infected with dsRed-expressing M. tuberculosis. Cells were fixed at the indicated time points and incubated with rabbit anti-Rab7, rabbit anti-cathepsin L, rabbit anti-LC3, and rat anti-LAMP2 antibodies, respectively. Alexa Fluor 405 goat anti-rabbit IgG (green) was used to detect Rab7, cathepsin L, and LC3. Alexa Fluor 647 goat anti-rat IgG (purple) was used to detect LAMP2. (B to F) The colocalization of MCVs with Rab7/LAMP2 (B), cathepsin L (C), cathepsin L/LAMP2 (D), LC3 (E), and LC3/LAMP2 (F) were quantified from 100 bacteria. Data are presented as mean ± SEM and analyzed by unpaired *t* test with a two-tailed *P* value: *, *P* < 0.05. All specimens were imaged with confocal microscopy and performed in triplicate. Values of *P < *0.05 were considered a statistically significant difference. Download FIG S8, PDF file, 0.9 MB. Copyright © 2021 Chen et al. 2021 Chen et al. https://creativecommons.org/licenses/by/4.0/ This content is distributed under the terms of the Creative Commons Attribution 4.0 International license.

10.1128/mSystems.00783-21.9 FIG S9 Mycobacterial load and histopathology analysis of lung and spleen at day 21 post infection. (A and B) Mycobacterial load in lung and spleen. Mice were infected with M. tuberculosis H37Ra (1 × 10^6^ CFUs) and then administered with aspirin at day 1 postinfection for 20 days. ETOH was used as infection control. Lungs and spleens were obtained at the indicated time points. CFU quantification was conducted on half of the homogenized lung and spleen. (C) H&E staining of lungs and spleens as indicated. The other half of the lung and spleen was fixed with buffed paraformaldehyde for H&E staining. (D) The histopathology of lungs and spleens at 21 days postinfection was analyzed with ImageJ and quantified by analyzing the number of inflammatory cells. (E) The histopathology of spleens was analyzed with ImageJ and quantified by analyzing the area of white pulps. Data are presented as mean ± SEM and analyzed by unpaired *t* test with two-tailed *P* values, with asterisks indicating statistically significant differences. Scale bars = 100 μm. Download FIG S9, PDF file, 28.7 MB. Copyright © 2021 Chen et al. 2021 Chen et al. https://creativecommons.org/licenses/by/4.0/ This content is distributed under the terms of the Creative Commons Attribution 4.0 International license.
