# Supplementary figures and images for: Distinct Persistence Fate of Mycobacterium tuberculosis in Various Types of Cells
Source: mSystems. 2021 Aug 17;6(4):e00783-21. doi: 10.1128/mSystems.00783-21 (PMC8409741; doi:10.1128/mSystems.00783-21)

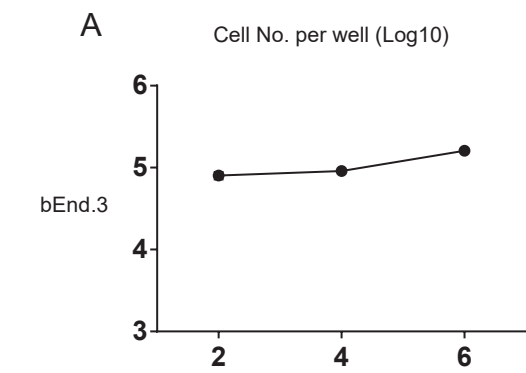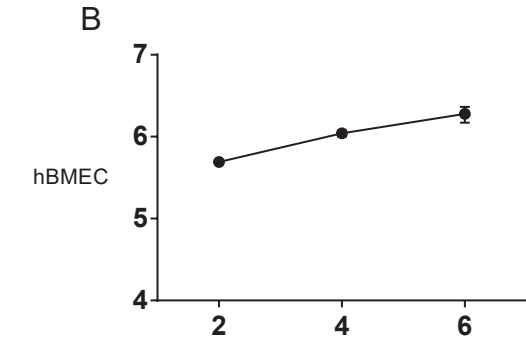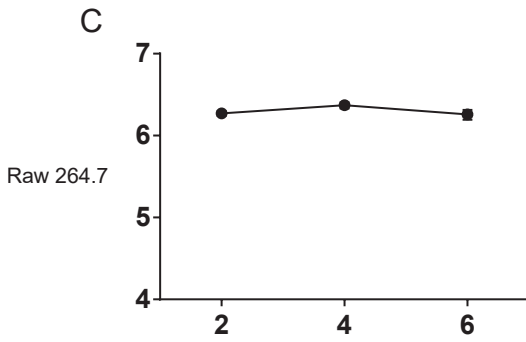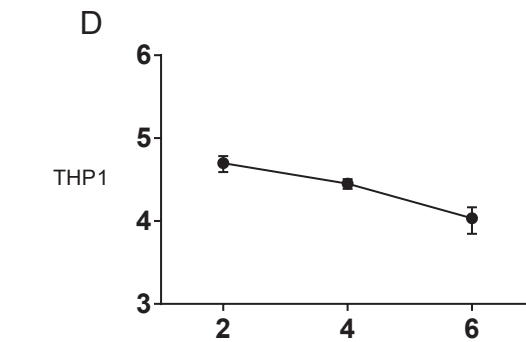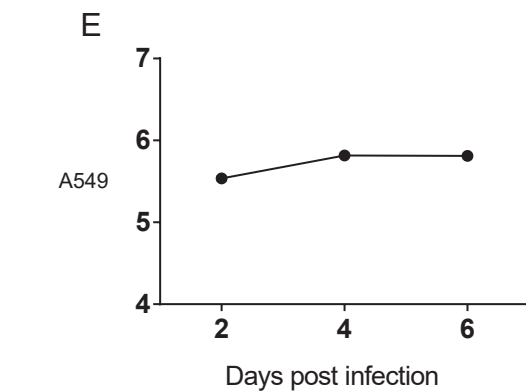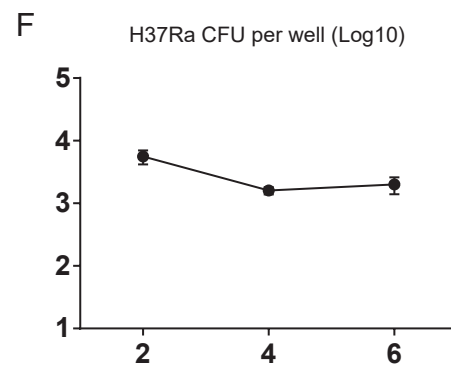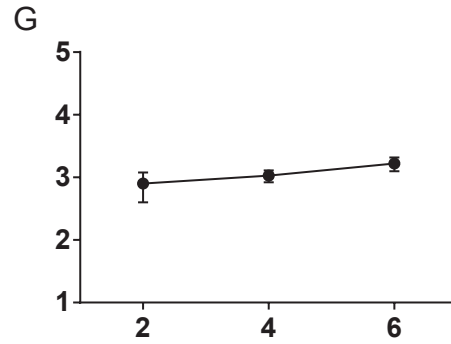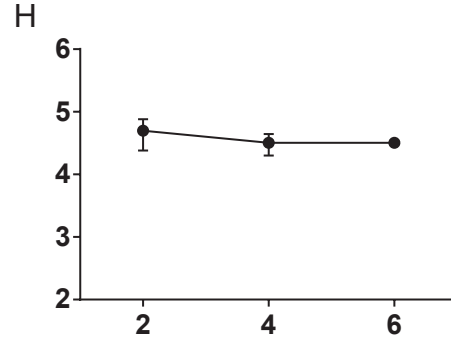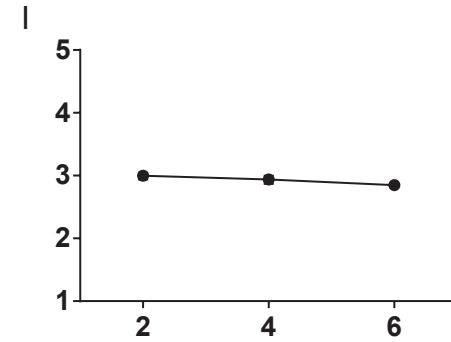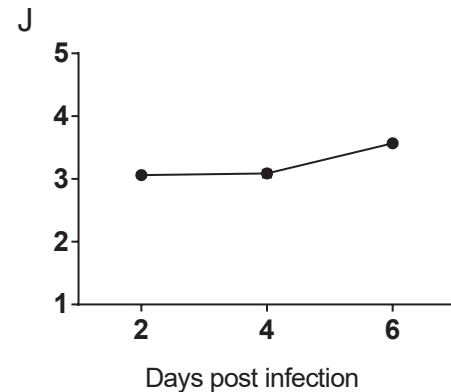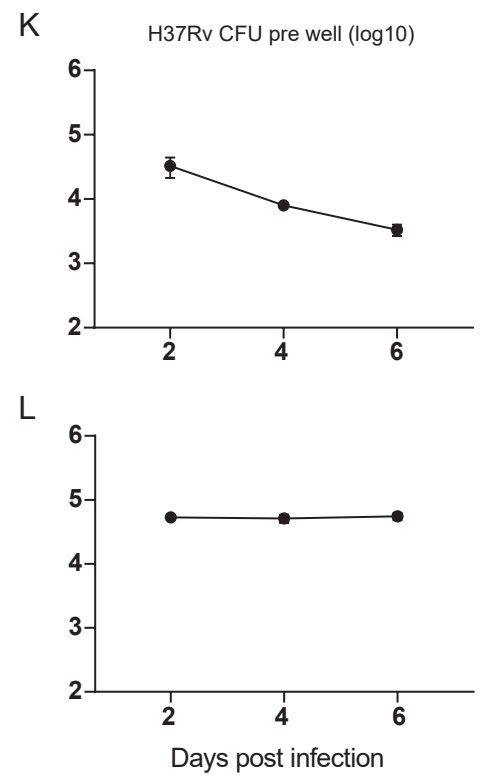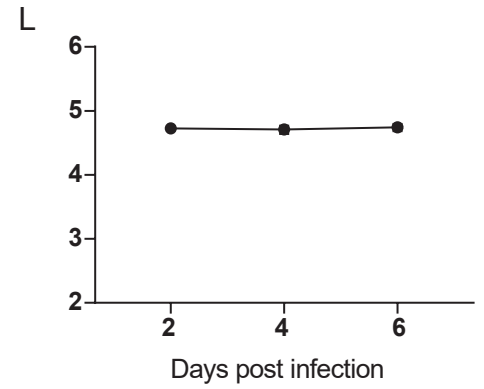

Supplement: FIG S1 [file msystems.00783-21-sf001.pdf]

# A hBMEC

day 0

day3

Rab5

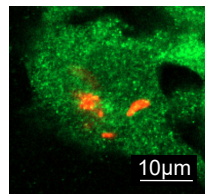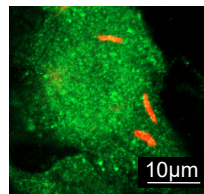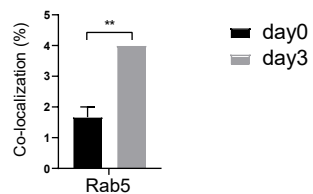

Rab7

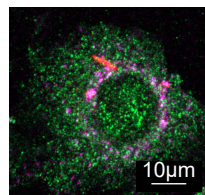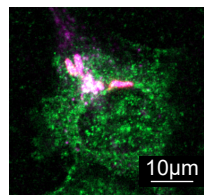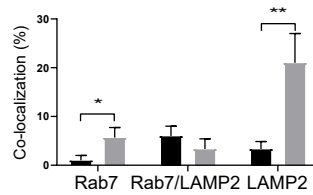

Cath.

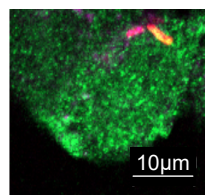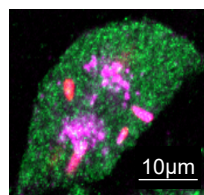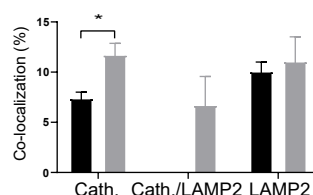

LC3

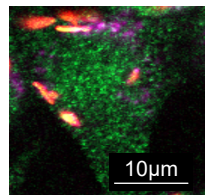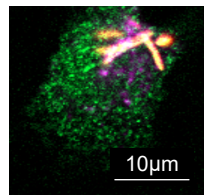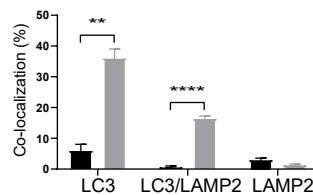

*M.tb* H37Rv

LAMP2

# B THP1

day 0

day3

Rab5

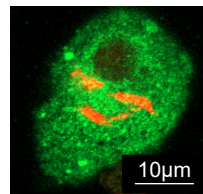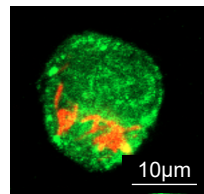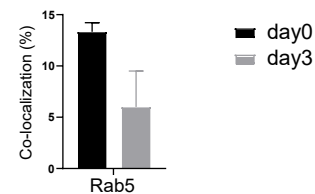

Rab7

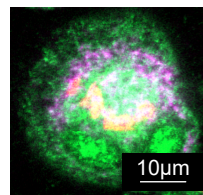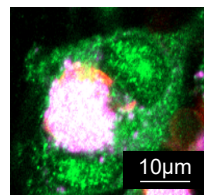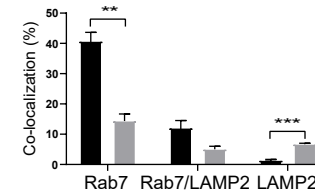

Cath.

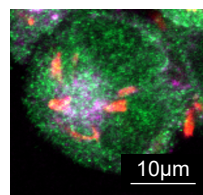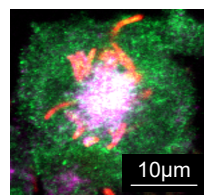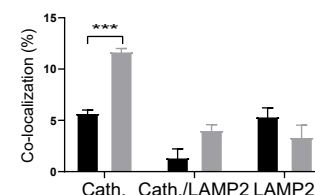

LC3

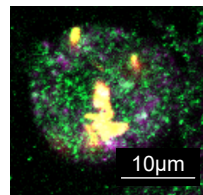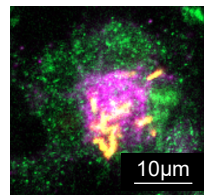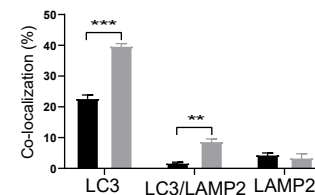

*M.tb* H37Rv

LAMP2

Supplement: FIG S2 [file msystems.00783-21-sf002.pdf]

A

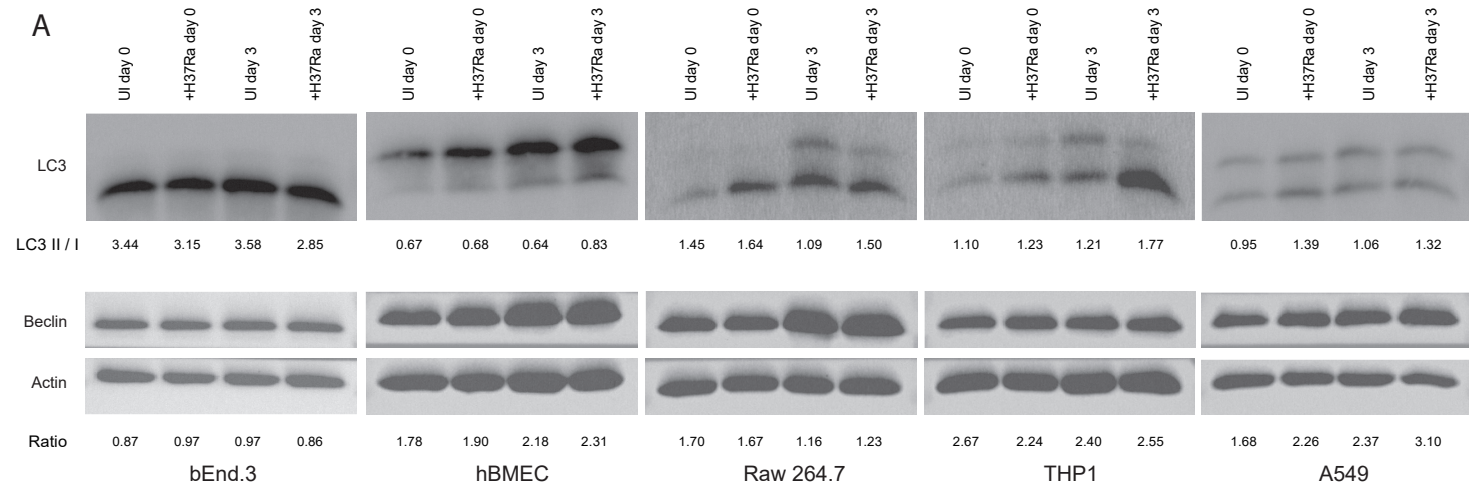

Supplement: FIG S3 [file msystems.00783-21-sf003.pdf]

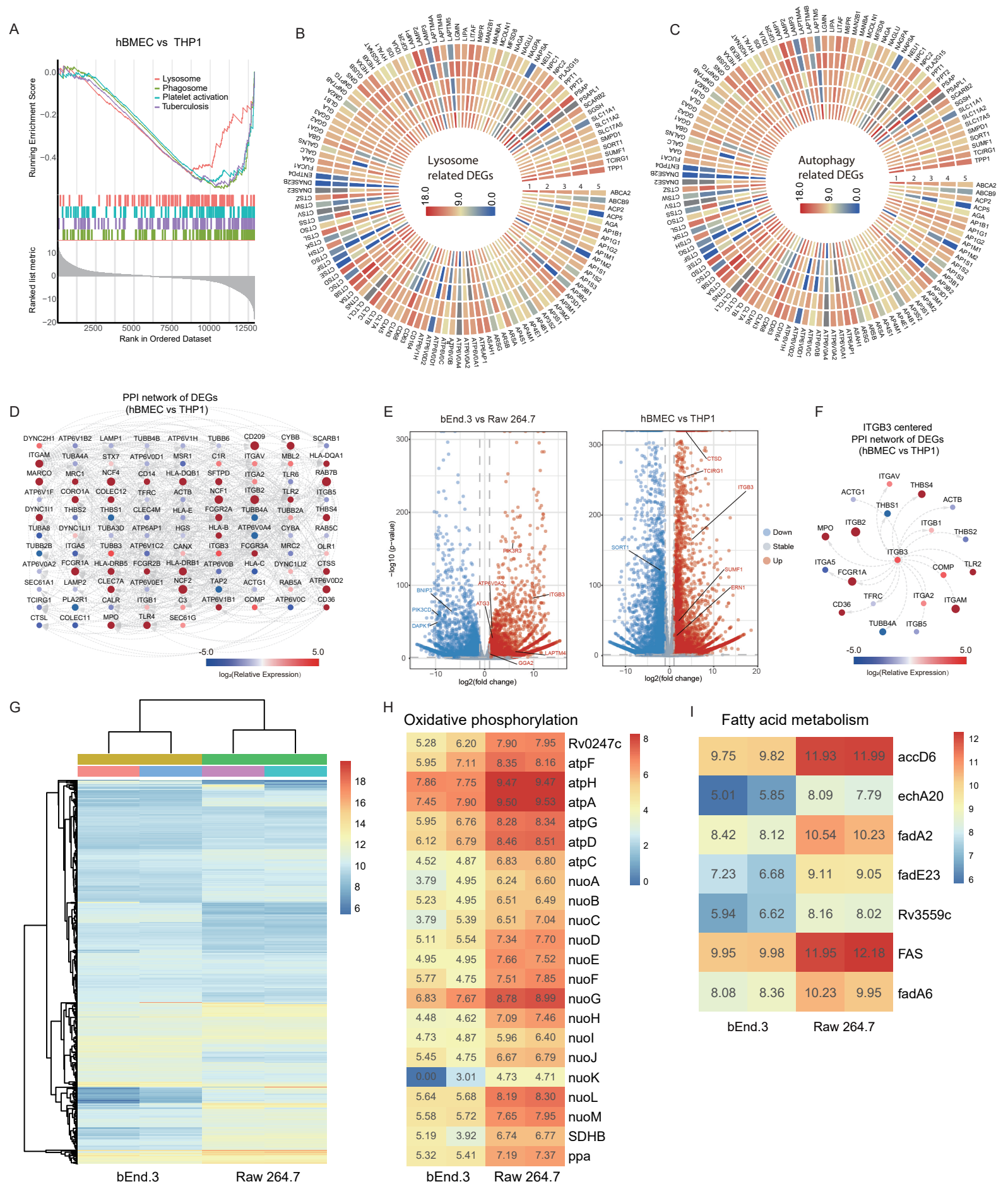

Supplement: FIG S4 [file msystems.00783-21-sf004.pdf]

A

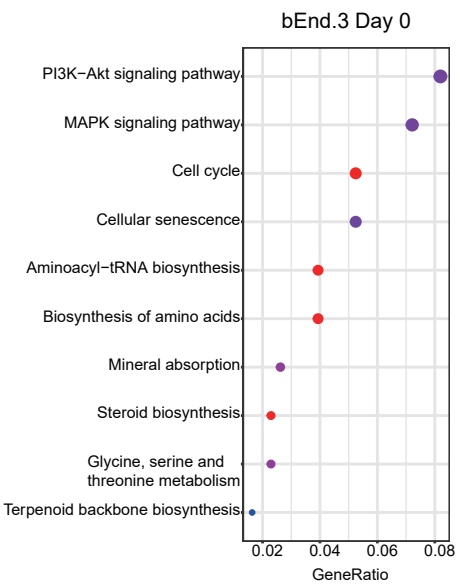

B

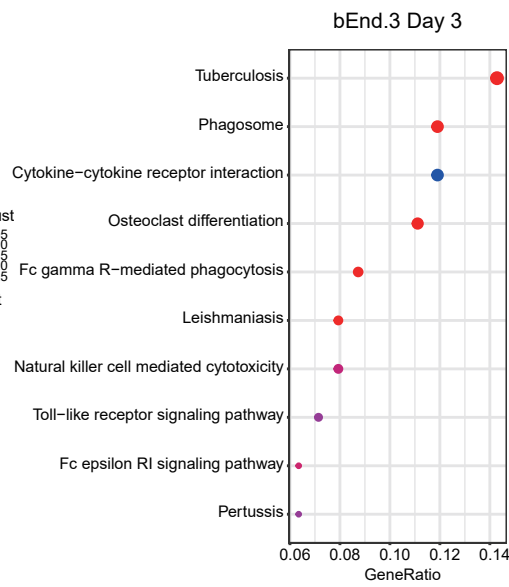

C

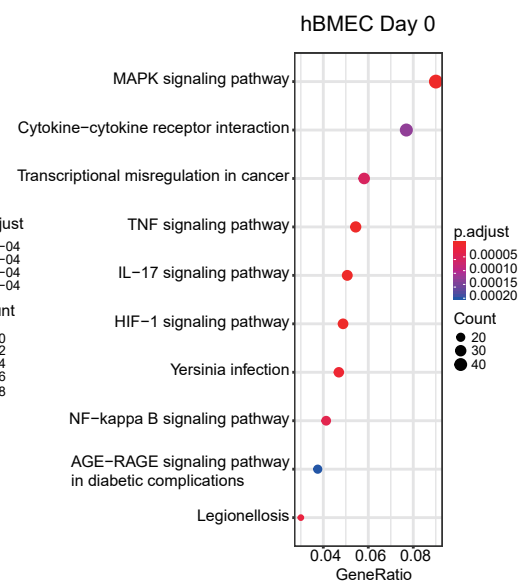

D

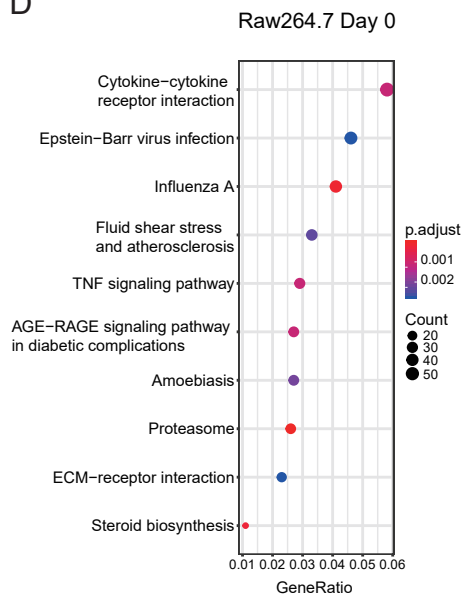

E

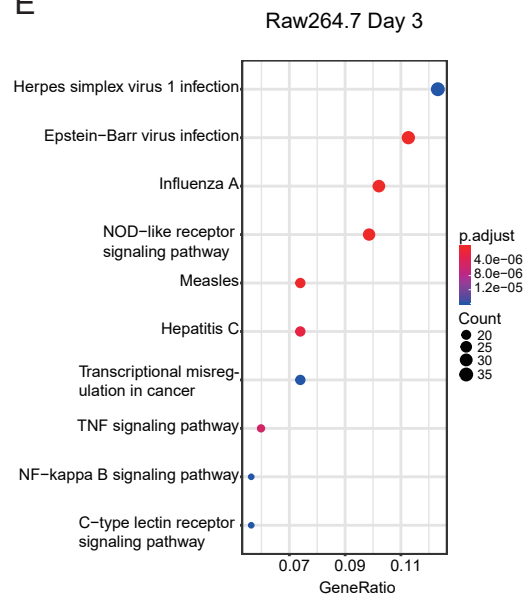

F

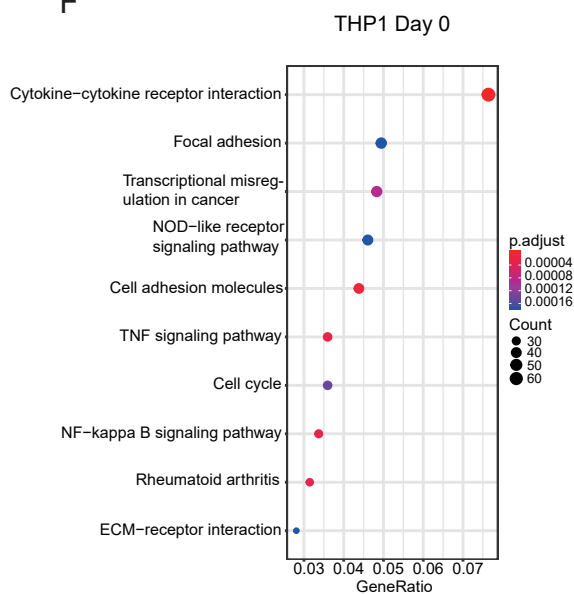

G

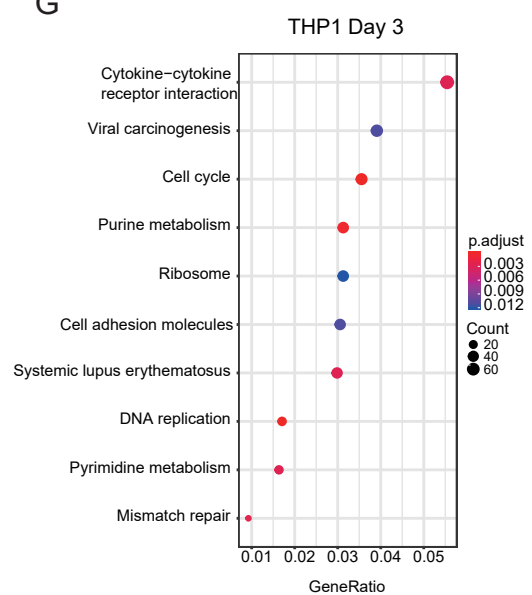

Supplement: FIG S5 [file msystems.00783-21-sf005.pdf]

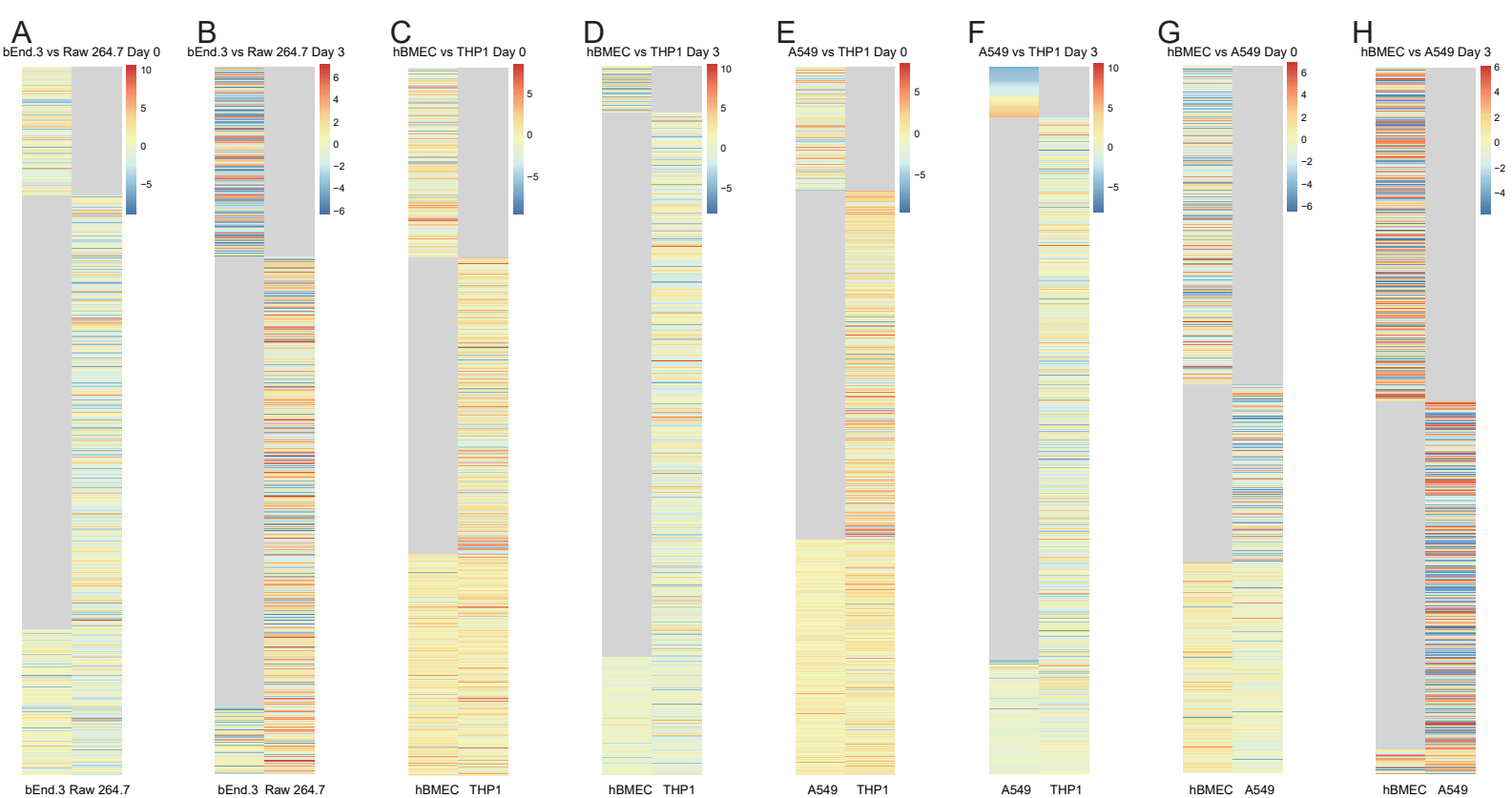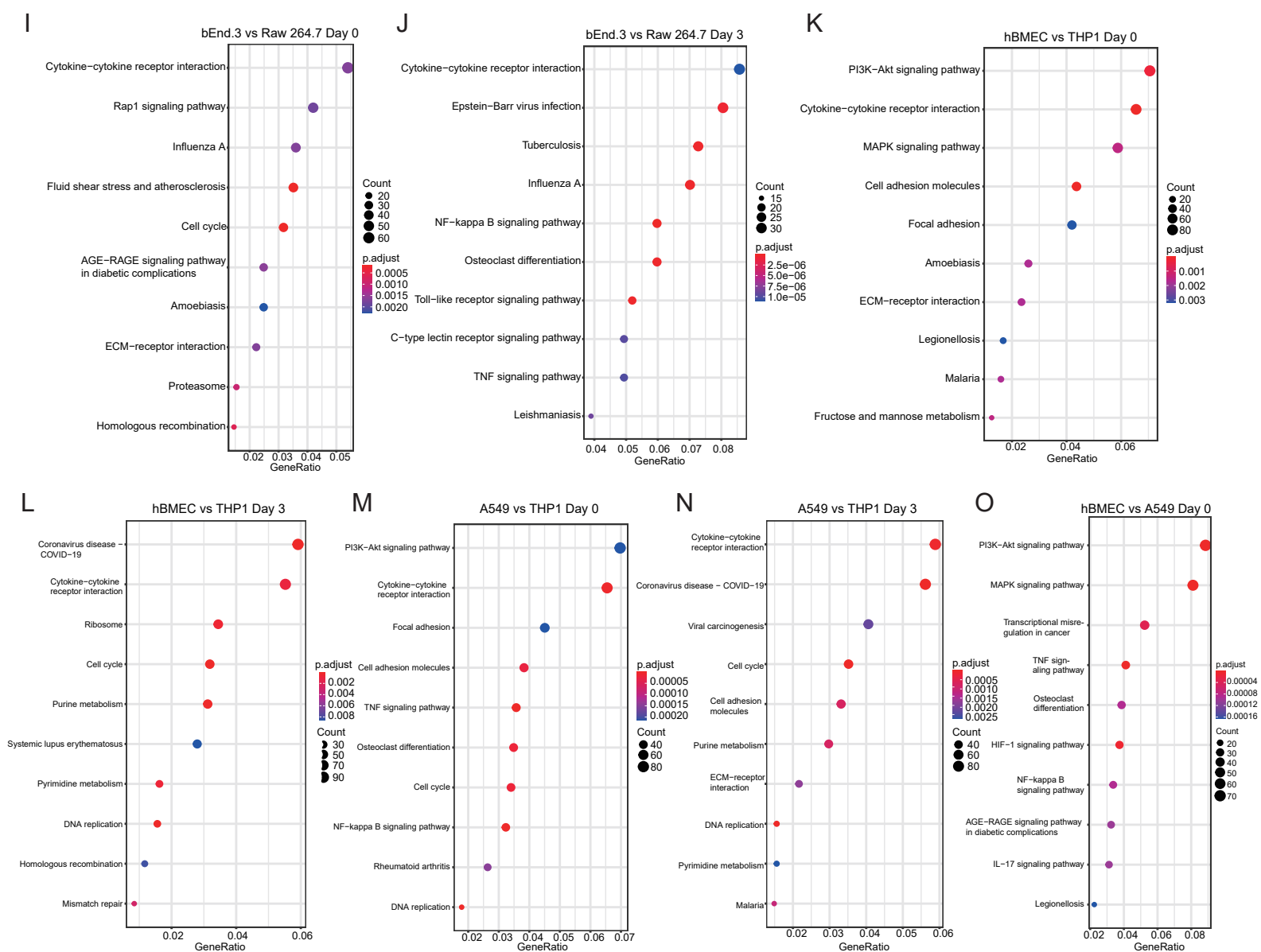

Supplement: FIG S6 [file msystems.00783-21-sf006.pdf]

A

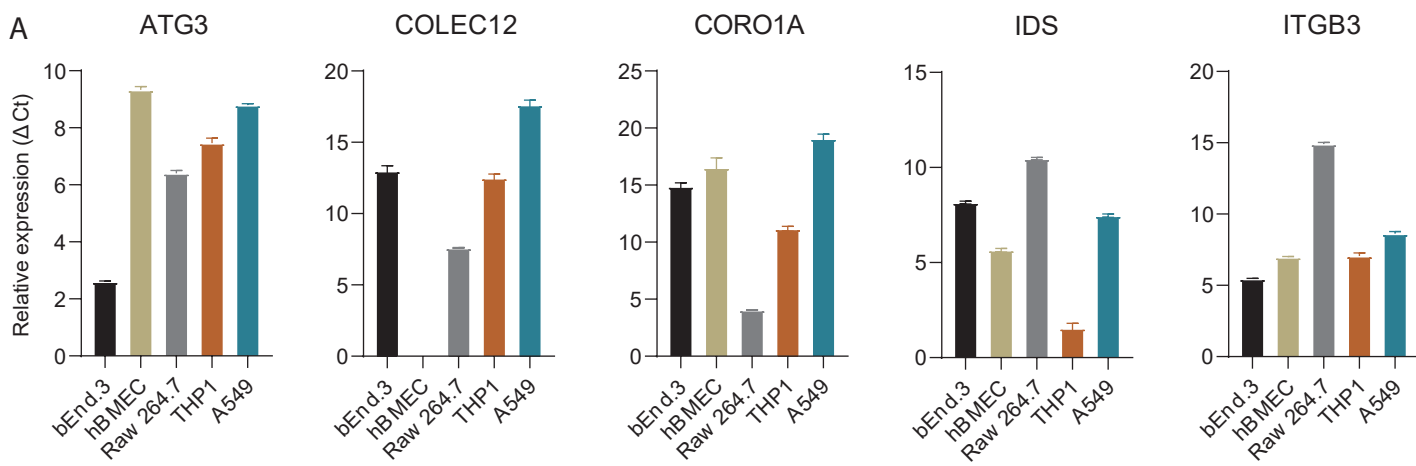

B

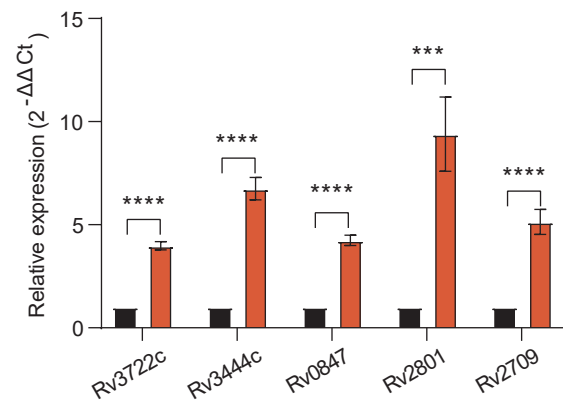

Supplement: FIG S7 [file msystems.00783-21-sf007.pdf]

**A**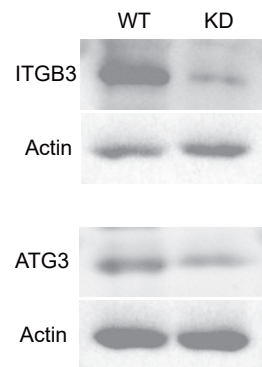**B**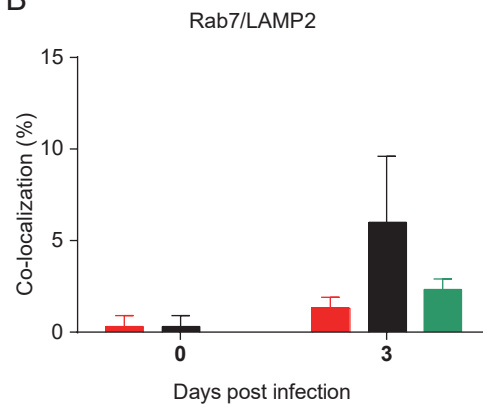**C**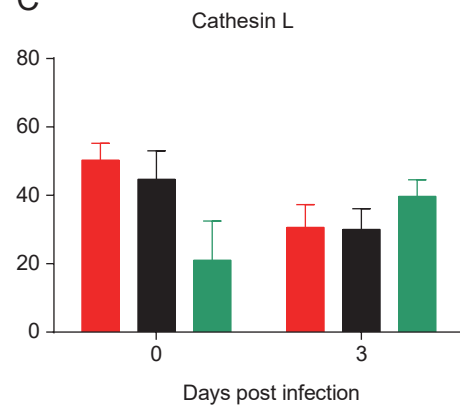**D**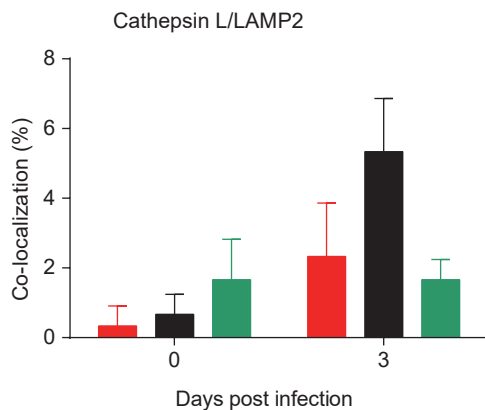**E**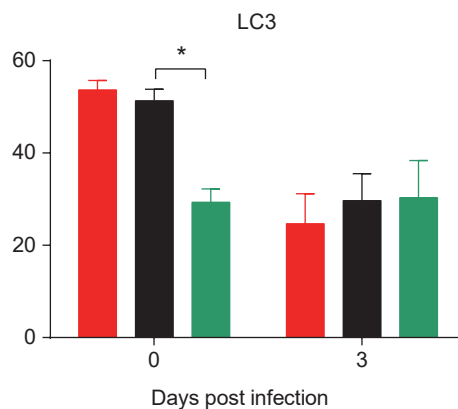**F**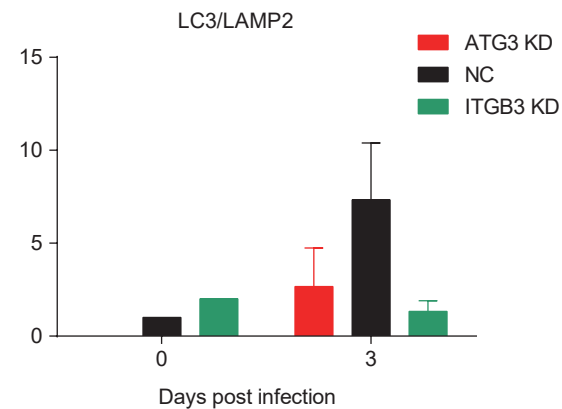

Supplement: FIG S8 [file msystems.00783-21-sf008.pdf]

A

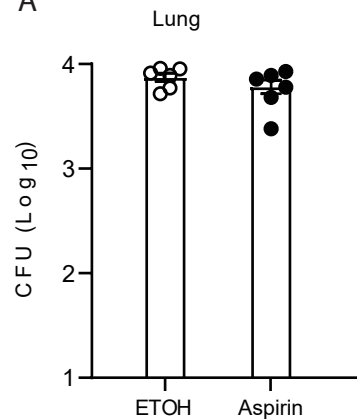

B

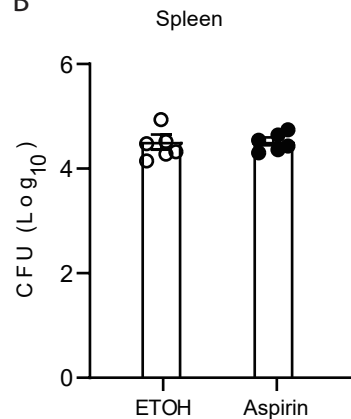

C

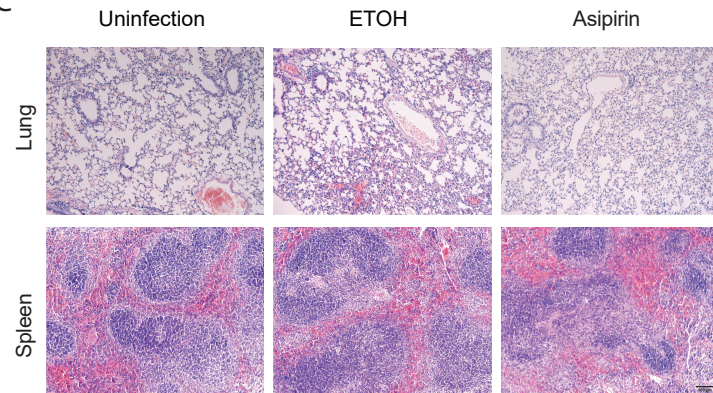

D

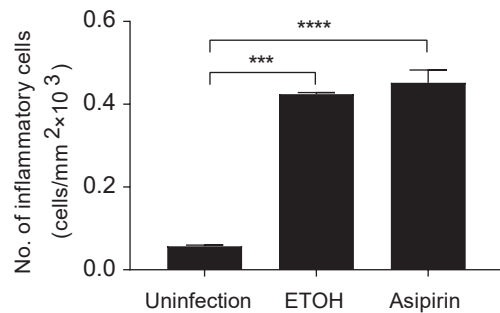

E

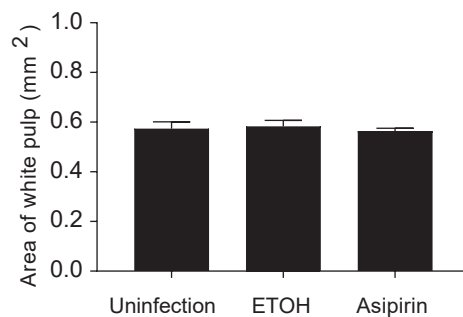

Supplement: FIG S9 [file msystems.00783-21-sf009.pdf]
